# Dual Targeting Approach Using 4‐Hydroxytamoxifen Neuropeptide Y Conjugates for Selective Addressing of Adipose Tissue

**DOI:** 10.1002/cmdc.202500668

**Published:** 2025-10-28

**Authors:** Anna Kohler, Eva‐Maria Jülke, Luke C. Darveniza, Jan Stichel, Annette G. Beck‐Sickinger

**Affiliations:** ^1^ Faculty of Life Sciences Institute of Biochemistry Leipzig University 04103 Leipzig Germany; ^2^ Molecular Imaging and Therapy Research Unit (MITRU) South Australian Health and Medical Research Institute (SAHMRI) 2 North Terrace Adelaide 5000 Australia

**Keywords:** liquid chromatography, mass spectrometry, peptide‐drug conjugates, receptors solid‐phase synthesis

## Abstract

Tamoxifen, a selective estrogen receptor modulator, reduces fat mass and induces adipose tissue browning in obese mice, suggesting its potential as an antiobesity drug. However, small‐molecule therapies often cause nonspecific side effects. The goal is to transport 4‐hydroxytamoxifen (4‐OHT) specifically into cells using the human neuropeptide Y (NPY) receptor type 1 (hY_1_R), which is highly expressed on the surface of adipocytes. NPY conjugates are generated that link 4‐OHT with three enzymatically cleavable and one self‐immolative diamine linkers. All conjugates exhibit similar behavior regarding receptor activation and internalization. The activity of the intracellularly released 4‐OHT is measured using a luciferase reporter gene assay. The diamine linker construct is the only conjugate that induces full reporter gene activation after internalization. However, the attachment of the drug to the peptide is unstable. Among peptides with enzymatically cleavable linkers, the conjugate with the GFLG linker exhibits the greatest reporter gene activity and is selected for further validation. A detailed analysis of its stability is performed using chromatography and mass spectrometry. Excellent plasma stability is demonstrated by fluorescence and isotopic labeling. These results demonstrate successful drug transport into target cells, paving the way for the further optimization of obesity therapies with reduced side effects.

## Graphical abstract

The [F^7^,P^34^]‐pNPY based peptide‐drug conjugate targets the Y_1_ receptor and induces internalization. Once inside the cell, 4‐hydroxytamoxifen is cleaved from the peptide and is released into the cytosol, where it activates the estrogen receptor *α*. This dual‐targeting mechanism aims to promote body weight loss and offers a potential therapeutic strategy for obesity and related metabolic disorders.

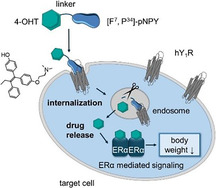

## 1 Introduction

For the successful treatment of chronic diseases such as obesity and cancer, it is important to have therapies with few side effects, as this can lead to a better prognosis. Most treatment options are based on small, active molecular drugs. However, because these drugs are distributed throughout the entire organism, only a small proportion of the administered active molecules reaches the target tissue. Consequently, adverse off‐target effects can diminish therapy efficacy and quality of life.^[^ ^1^ ^,^ ^2^ ^]^ Peptide‐drug conjugates (PDCs) are a promising approach to reducing these side effects. In PDCs, the drug is transiently attached to the peptide, enabling specific transport of the drug into target cells. Unlike antibody‐drug conjugates, these peptides have several advantages. They can reach the target tissue more effectively, pose a lower risk of activating the immune response, and can address different targets with versatility.^[^ ^3^ ^]^ G protein‐coupled receptors (GPCRs) are easily accessible targets for PDCs because they are located in the cell membrane. Although ≈35% of FDA‐approved drugs target GPCRs, their full therapeutic potential has yet to be realized due to their involvement in various physiological processes.^[^ ^4^ ^,^ ^5^ ^]^ A well‐studied GPCR family is the human neuropeptide Y receptors (hYR), which consists of the four members hY_1_R, hY_2_R, hY_4_R, and hY_5_R. These receptors are activated by three distinct peptide ligands: neuropeptide Y (NPY), pancreatic polypeptide (PP), and peptide YY (PYY), each with different affinities. Together, these receptors and peptides form a multiligand/multireceptor system, which is involved in many physiological pathways such as food intake or gastric secretion.^[^ ^6^ ^,^ ^7^ ^]^ It has been widely documented that the Y_1_R is expressed in most breast cancers and breast cancer‐derived metastases.^[^ ^8^ ^,^ ^9^ ^]^ High levels of Y_1_R are also present in murine and human adipose tissue, making this receptor an interesting target for improved obesity therapies.^[^ ^10^, ^11^ ^–^ ^12^ ^]^ The peptide that demonstrates the strongest affinity for hY_1_R is NPY. This peptide consists of 36 amino acids and was first identified by Tatemoo et al.^[^ ^13^ ^]^ Based on this native ligand, hY_1_R specific PDCs for therapeutic approaches have been investigated in detail with the hY_1_R‐ selective peptide [F^7^,P^34^]‐NPY.^[^ ^14^, ^15^, ^17^ ^–^ ^18^ ^]^ Importantly, a modification of this peptide at Lys^4^ is possible without significantly affecting receptor binding behavior.^[^ ^16^ ^,^ ^19^, ^20^ ^–^ ^21^ ^]^

In general, binding of the corresponding peptide ligand to the receptor triggers an intracellular signaling cascade, which, in addition to G protein activation and its downstream effectors, also results in the internalization of the receptor–peptide complex. In the context of PDCs, smart linker systems, including the frequently used Gly‐Phe‐Leu‐Gly (GFLG) and Val‐Cit (VCit) linkers, have been applied to temporarily couple the payload to the peptide. This ensures subsequent uptake of the drug into target cells.^[^ ^22^ ^]^ In the endosome, enzymes such as cathepsin B cleave the linkers, allowing the drug to diffuse through the endosomal membrane into the cytosol and bind to its intracellular target.^[^ ^14^ ^,^ ^23^, ^24^, ^25^ ^–^ ^26^ ^]^ There are also various other linker systems that can be cleaved through other processes, such as self‐immolative diamine linkers, which use pH changes.^[^ ^27^ ^,^ ^28^ ^]^ In this study, we aimed to develop PDCs for treating obesity. Tamoxifen, a selective estrogen receptor modulator used in the standard treatment of ER‐positive breast cancer, is a promising drug candidate. Experimental data suggest that tamoxifen can protect against weight gain and adipose tissue accumulation.^[^ ^29^ ^,^ ^30^ ^]^ Studies in obese mice have shown improved body composition and adipose tissue biology after six weeks of tamoxifen treatment, as well as browning of subcutaneous adipose tissue.^[^ ^31^ ^]^ However, high doses of tamoxifen have been shown to promote diabetes, likely due to increased hepatic fat. It has been hypothesized that acute adipose depletion impairs compensatory capacity during weight regain, resulting in ectopic fat accumulation, steatosis, and insulin resistance.^[^ ^32^ ^]^ Our objective is to selectively target Y_1_R‐expressing cells using Y_1_R agonistic peptides to reduce these side effects; then, tamoxifen will only accumulate in adipocytes. To facilitate covalent attachment to the peptide, we used the more potent metabolite, 4‐hydroxytamoxifen (4‐OHT).^[^ ^33^ ^,^ ^34^ ^]^ The biochemical behavior of several PDCs was validated in various artificial cell‐based assays.

## 2 Results and Discussion

### 2.1 Synthesis of Peptide Conjugates with 4‐OHT Using SPPS

**Figure 1:**
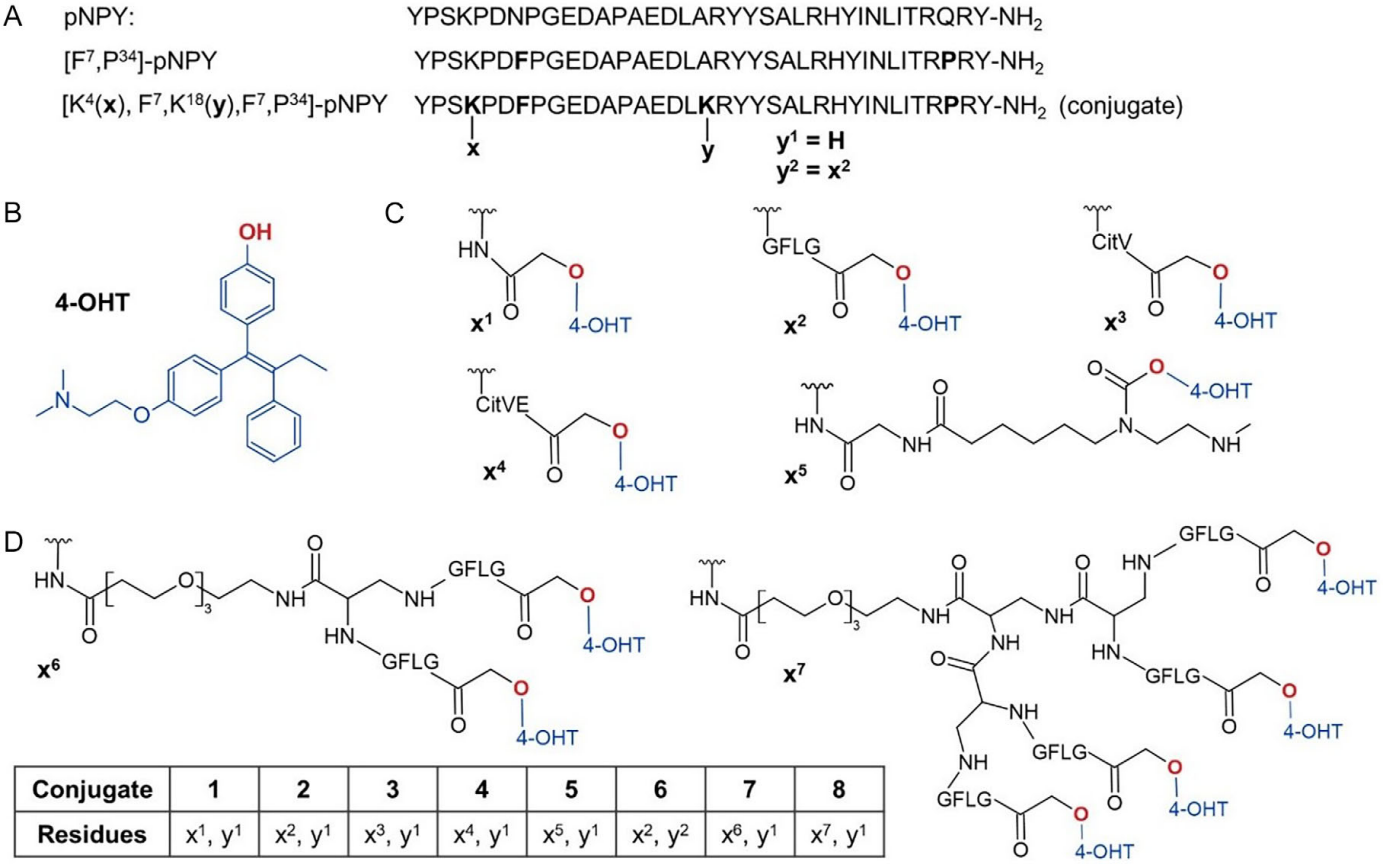
Peptide design. A) The amino acid sequence of porcine neuropeptide Y (pNPY), the Y_1_R selective [F^7^,P^34^]‐pNPY analog and its positions for drug attachments are shown. B) Chemical structure of 4‐hydroxytamoxifen (4‐OHT). C) Different linker structures for 4‐OHT conjugates are shown: *x* ^1 ^= direct drug attachment without cleavable linker, *x* ^2 ^= GFLG linker, *x* ^3 ^= CitV linker, *x* ^4 ^= CitVE linker, and *x* ^5 ^= selfimmolative diamine linker. D) Branched peptide conjugates with two or four 4‐OHT molecules per shuttle.

PDCs enable the specific delivery of active small‐molecule drugs into target cells through selective binding to surface receptors such as GPCRs. In this study, various cleavable linker systems and different payload numbers have been used. All conjugates **1–8** (**Figure** **1**) were synthesized with a combination of automated and manual solid‐phase peptide synthesis (SPPS) using the fluorenyl methoxycarbonyl/tert‐butyl (Fmoc/tBu) strategy.

**Figure 2:**
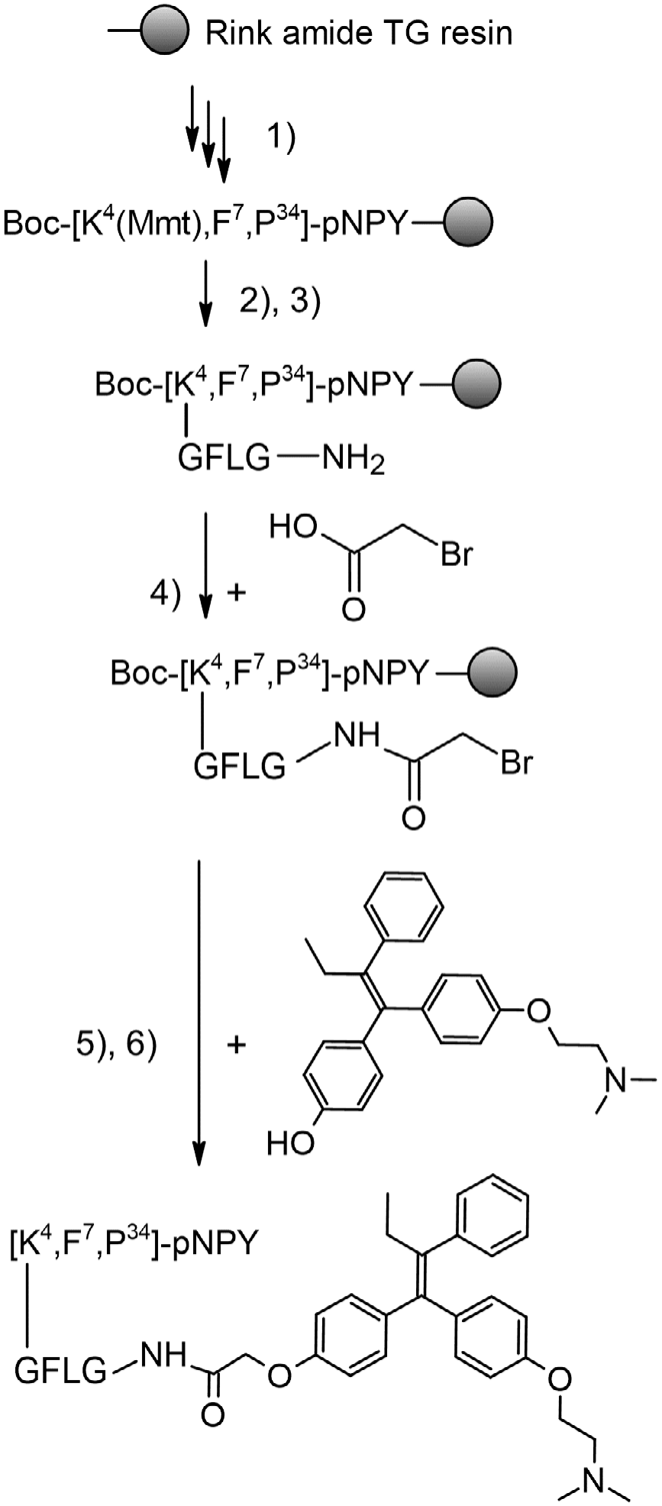
Synthesis strategy for 4‐OHT conjugates exemplified by the conjugate with a GFLG linker. 1) SPPS of the peptide backbone using Fmoc/tBu strategy on a tentagel (TG) resin. 2) Selective methoxytrityl (Mmt) cleavage with 2% TFA, 5% triisopropylsilane (TIS) in DCM; 15 × for 1 min. 3) Successive manual coupling of Gly, Phe, Leu, Gly with DIC and HOBt in DMF (5 equiv. each), 3–16 h. 4) Coupling of bromoacetic acid with DIC and HOBt in DMF (5 equiv. each), 3 h. 5) Coupling of 4‐OHT (1.5 equiv) using K_2_CO_3_ (3 equiv) in anhydrous THF, argon atmosphere at 40 °C for 3 h. 6) Full cleavage with 90% TFA, 7% thioanisole (TA) and 3% 2,2‐(ethylendioxy)‐diethanthiol (DODT).

Since tamoxifen lacks a functional group that would allow it to attach to the peptide, the potent metabolite 4‐hydroxy‐tamoxifen (4‐OHT) was used instead. Its hydroxyl group was then used to attach the drug to the peptide (**Figure** **2**). Standard coupling strategies with, e.g., N, N‐diisopropylcarbodiimide (DIC) and 1hydroxybenzotriazole (HOBt) have not been successful. Instead, drug coupling by the hydroxyl group was tried with potassium carbonate (K_2_CO_3_) in N, N‐dimethylformamide (DMF) at 50 °C with a halogen as a leaving group, as previously published.^[^ ^35^ ^]^ K_2_CO_3_ with anhydrous tetrahydrofuran (THF) at 40 °C led to a higher reaction efficiency (see Figure S1, supporting information). By using anhydrous DMF, the coupling efficiency of 22.1% was achieved, whereas the use of anhydrous THF led to an increased yield of 34.6%. Isomerization of 4‐OHT during synthesis caused the final peptide to elute as a double peak in preparative reversed‐phase high‐performance liquid chromatography (RP‐HPLC). The structure of 4‐OHT and all different synthesized conjugates **1–8** are shown in Figure 1. By introducing double Fmoc‐protected diamino‐propionic acid (Fmoc‐L‐Dap(Fmoc)‐OH) to the free N*ε*‐amino group of Lys^4^ or Lys^18^, branching was achieved. Following Fmoc cleavage, both free amino groups of the original lysine side‐chain were elongated simultaneously during SPPS. However, the synthesis of multiply loaded conjugates **6–8** proved to be challenging. Due to the isomerization of 4‐OHT, multiple product peaks appeared for multiloaded peptides, making the separation of unwanted by‐products difficult. Additionally, the solubility of the multiloaded peptides decreased significantly with every further addition of the hydrophobic 4‐OHT. Nevertheless, sufficiently purified peptides were obtained and compared based on their ability to release 4‐OHT functionally. A summary of all the peptides and conjugates, along with their final purity analytics, is listed in Table S1 in the supplementary section.

### 2.2 4‐OHT‐Peptide Conjugates are Potent hY_1_R Agonists

**Figure 3:**
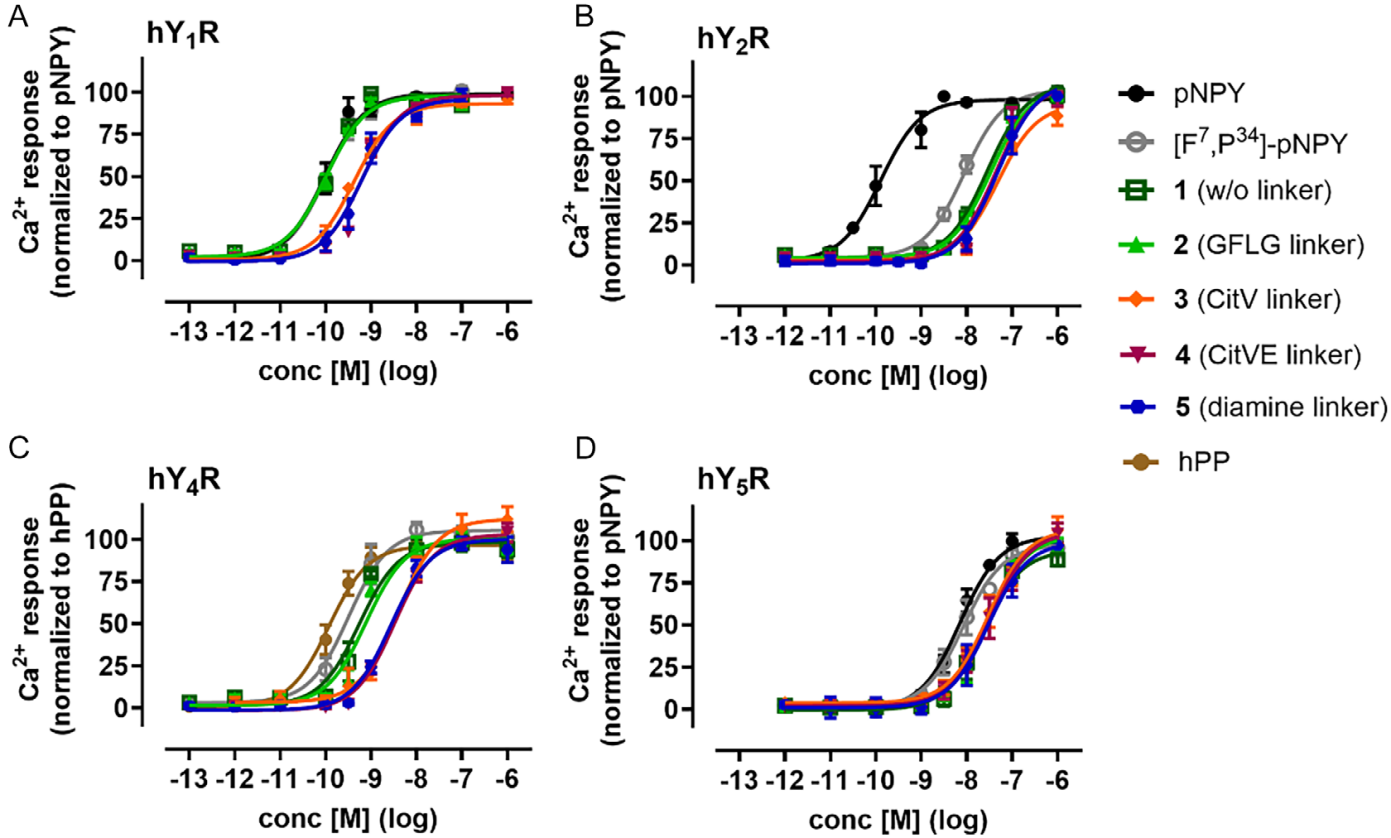
Receptor activation of 4‐OHT‐conjugates compared to the native ligands and unmodified [F^7^,P^34^]‐NPY. In stably transfected COS7cells expressing Δ6G*α*_qi4‐myr_ and A) hY_1_R, B) hY_2_R, C) hY_4_R, or D) hY_5_R, G‐protein activation was tested by using an Ca^2+^‐flux assay. Sigmoidal curves were achieved by stimulation with increasing peptide concentrations. Data were normalized to the corresponding native ligand (NPY for hY_1_R, hY_2_R, hY_5_R, and hPP for hY_4_R). Data are shown as mean ± SEM from *n *≥ 2 independent experiments performed with technical triplicates.

The influence of the linker and 4‐OHT‐attachments on receptor activation was investigated by a calcium‐flux assay (**Figure** **3**, **Table** **1**). All conjugates exhibited excellent activity at the hY_1_R receptor, with EC_50_ values in the lower nanomolar range. Conjugates **1** and **2** showed the best receptor activation, with an EC_50_ value of 0.1 nM, corresponding to that of the native ligand, NPY. Since the conjugates share the [F^7^,P^34^]‐NPY backbone, they all showed a significantly reduced potency at the hY_2_R, with EC_50_ shifts ranging from 67‐ to 341‐fold, compared to the hY_1_R. Therefore, all conjugates are highly selective for the hY_1_R over the hY_2_R. Conjugate **2** showed the strongest EC_50_ shift here as well. Interestingly, all conjugates also activated the hY_4_R with EC_50_ values increased only by 4‐ to 10‐fold, which are still in the low nanomolar range. This coactivation is primarily due to the high sequence homology between the two subtypes.^[^ ^36^ ^,^ ^37^ ^]^ However, in comparison to the unmodified [F^7^,P^34^]‐NPY, which had an EC_50_ value of 0.3 nM at hY_4_R, the drug attachment to the peptide resulted in a slightly improved selectivity.

**Table 1:** Potency of 4‐OHT‐conjugates with different cleavable linker structures compared to the native ligands and unmodified [F^7^,P^34^]‐NPY at different Y receptors in a calcium flux assay performed in stably transfected COS‐7‐hY_1/2/4/5_R‐Δ6G*α*_qi4‐myr_‐cells. Data were obtained from at least two independent experiments performed in triplicates. EC_50_‐values and pEC_50 _± SEM are given for each conjugate.

EC_50_ values [nM] (pEC_50 _± SEM)				
	hY_1_R	hY_2_R	hY_4_R	hY_5_R
pNPY/ hPP	0.1 (10.0 ± 0.08)	0.1 (9.9 ± 0.12)	0.1 (9.9 ± 0.08)	7.2 (8.2 ± 0.07)
[F^7^,P^34^]‐pNPY	0.1 (10.0 ± 0.08)	8.8 (8.1 ± 0.04)	0.3 (9.5 ± 0.10)	8.5 (8.1 ± 0.09)
1 (w/o linker)	0.1 (10.0 ± 0.07)	28.4 (7.6 ± 0.08)	0.6 (9.3 ± 0.10)	22.9 (7.6 ± 0.11)
2 (GFLG linker)	0.1 (10.0 ± 0.05)	34.1 (7.5 ± 0.05)	0.8 (9.1 ± 0.09)	28.3 (7.6 ± 0.09)
3 (CitV linker)	0.4 (9.4 ± 0.06)	48.6 (7.3 ± 0.10)	4.1 (8.4 ± 0.12)	29.8 (7.5 ± 0.08)
4 (CitVE linker)	0.7 (9.2 ± 0.07)	43.7 (7.4 ± 0.12)	3.4 (8.5 ± 0.06)	32.8 (7.5 ± 0.09)
5 (diamine linker)	0.6 (9.2 ± 0.09)	48.4 (7.3 ± 0.13)	2.8 (8.6 ± 0.07)	29.9 (7.5 ± 0.19)

Similar to unmodified NPY and [F^7^,P^34^]‐NPY, the EC_50_ values measured on hY_5_R are considerably higher than those measured on hY_1_R for all conjugates. These results from the short‐term response in the calcium level have been validated in an IP1 accumulation assay (see Figure S2).

### 2.3 4‐OHT‐Conjugates Induce Receptor Internalization and Intracellular Effects

**Figure 4:**
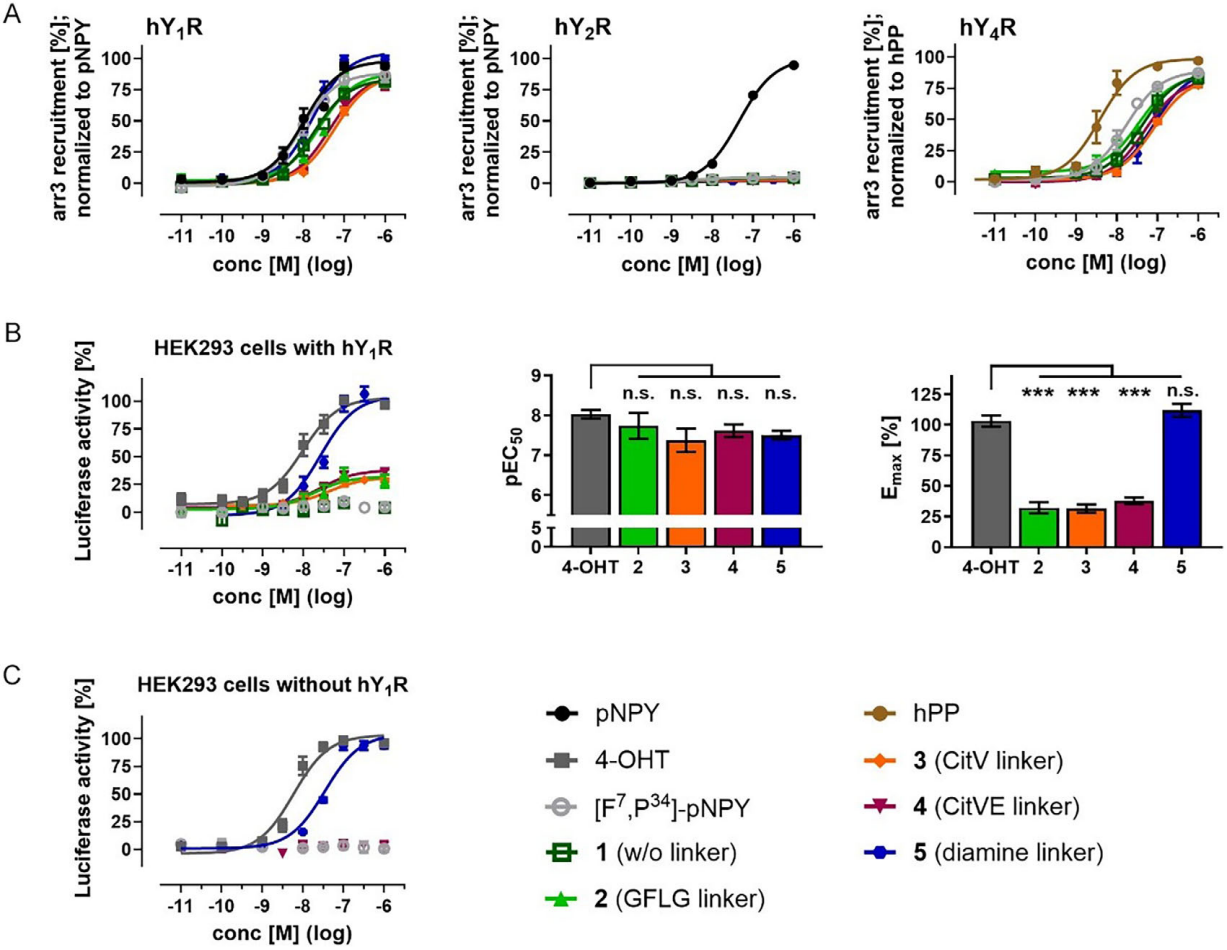
Peptide‐induced intracellular effects. A) Arrestin‐3 (arr3) recruitment to hY_1/2/4_R was validated with a BRET‐based arr3 recruitment assay 15 min after stimulation. Transiently transfected HEK293 cells expressing hY_1/2/4_R‐eYFP and Nluc‐arr3 were stimulated with increasing peptide concentrations. Values were normalized to the native ligands (NPY for hY_1_R and hY_2_R, hPP for hY_4_R). Data points represent mean ± SEM from *n *≥ 2 independent experiments with technical triplicates. Luciferase reporter gene assay in transiently transfected HEK293‐cells expressing hY_1_R‐eYFP. B) ER*α*‐pEGFP and TATA‐luciferase. Cells were stimulated with increasing concentration of 4‐OHT, [F^7^,P^34^]‐NPY, or peptide‐conjugates for 24 h. Values were normalized to 4‐OHT. Data points represent mean ± SEM from *n *≥ 3 independent experiments with technical triplicates. Calculated pEC_50_‐ and E_max_‐values are shown as bar graphs. Dunnnet's 1‐ way ANOVA test was used to determine statistical significance; *, *p* < 0.033; **, *p* < 0.002; ***, *p* < 0.001, n.s. = not significant. C) Luciferase reporter gene assay in transiently transfected HEK293‐cells only expressing ER*α*‐pEGFP and TATA‐luciferase.

Many GPCRs recruit arrestin‐3 (arr3) after agonist binding and subsequently undergo internalization. We used a highly sensitive NanoBRET‐based arr3 recruitment assay to test for peptide‐induced internalization 15 min after stimulation.^[^ ^38^, ^39^ ^–^ ^40^ ^]^ Compared to the native ligand NPY, both [F^7^,P^34^]‐NPY and all conjugates show similar EC_50_ values in the nanomolar range, which were only slightly higher than the value obtained for NPY (EC_50_ = 10.1 nM, **Figure** **4**A, **Table** **2**). Arr3‐mediated internalization at the hY_2_R following stimulation could be excluded for all conjugates. In contrast, arr3 recruitment was also detected at hY_4_R, albeit with a higher EC_50_ value. This coactivation is consistent with the receptor activation data. As the hY_5_R is known to show limited internalization, this receptor was not studied.^[^ ^41^ ^]^ Despite the coactivation and internalization of the hY_4_R by conjugate binding, the selectivity is enhanced compared to that of free 4‐OHT. Additionally, the specificity of tamoxifen for the ER*α* minimizes side effects further. Using this dual‐targeting approach, the conjugates only become active in cells that take up the drug by hY_1_R or hY_4_R and also express ER*α* intracellularly. hY_4_R is predominantly expressed in the gastrointestinal tract, but also in the brain, coronary artery, pancreas, and prostate.^[^ ^42^, ^43^ ^–^ ^44^ ^]^ In contrast, ER*α* is mainly expressed in female tissue and to a lesser extent in adipose tissue, brain, bone, prostate, and liver.^[^ ^45^ ^]^ Furthermore, uptake into hY_4_Rexpressing cells could be minimized by a smart application strategy of the drug conjugates. Peptides are typically rapidly degraded by gastric enzymes, thereby limiting their oral bioavailability; however, there are a few exceptions.^[^ ^46^ ^]^ Thus, the most promising approach for a successful therapy would be a subcutaneous application into adipose tissue. Consequently, the coactivation of the hY_4_R by the 4‐OHT conjugates is a minor issue, because our approach focuses on adipocytes that exclusively express hY_1_R.

**Table 2:** Intracellular effects of 4‐OHT‐conjugates in nanoBRET‐based arrestin‐3 recruitment assay and luciferase reporter gene assay were confirmed in transiently transfected HEK293 cells. Data was obtained from at least three independent experiments performed in triplicates (arrestin‐3 recruitment) or quadruplicates (luciferase reporter gene). EC_50_‐values and pEC_50 _± SEM are listed.

	Arrestin‐3 recruitment			Luciferase reporter gene assay	
	EC_50_ values [nM] (pEC_50 _± SEM)			EC_50_ values [nM] (pEC_50 _± SEM)	
	hY_1_R	hY_2_R	hY_4_R	hY_1_R	w/o hY_1_R
4‐OHT	–	–	–	9.4 (8.0 ± 0.11)	5.8 (8.2 ± 0.10)
pNPY/ hPP	10.1 (8.0 ± 0.08)	45.8 (7.3 ± 0.02)	3.9 (8.4 ± 0.10)	–	–
[F^7^,P^34^]‐pNPY	8.7 (8.1 ± 0.08)	n. d.a)	16.9 (7.8 ± 0.08)	n. d.^a)^	n. d.a)
1 (w/o linker)	18.1 (7.7 ± 0.09)	n. d.a)	41.7 (7.4 ± 0.11)	n. d.^a)^	not tested
2 (GFLG linker)	25.0 (7.6 ± 0.12)	n. d.a)	34.9 (7.5 ± 0.11)	18.4 (7.7 ± 0.32)	not tested
3 (CitV linker)	57.4 (7.2 ± 0.06)	n. d.a)	80.4 (7.1 ± 0.08)	42.1 (7.4 ± 0.29)	not tested
4 (CitVE linker)	43.1 (7.4 ± 0.08)	n. d.a)	48.9 (7.3 ± 0.10)	24.4 (7.6 ± 0.16)	n. d.a)
5 (diamine linker)	16.8 (7.8 ± 0.09)	n. d.a)	83.1 (7.1 ± 0.10)	26.3 (7.6 ± 0.11)	34.8 (7.5 ± 0.10)

a) could not be determined

Next, we investigated the functional binding of 4‐OHT to ER*α* following the internalization of the peptide‐drug conjugate using a luciferase reporter gene assay. Therefore, we tested HEK293 cells that transiently express hY_1_R, ER*α*, and an ER*α* activity‐dependent luciferase. ER*α* forms dimers and translocates into the nucleus upon activation, where it binds to the estrogen response element (ERE) upstream of the luciferase gene and induces luciferase expression. As a positive control, unbound 4‐OHT exhibited a concentration‐dependent induction of luciferase expression with an EC_50_ value of 9.4 nM (Figure 4B, Table 2). Conjugates **2–4**, which have enzymatically cleavable linkers, induced luciferase activity with only slightly increased EC_50_ values ranging from 18.4 to 42.1 nM. However, conjugates **2–4** induced a significantly reduced maximum activity of ≈30%. Neither the unmodified [F^7^,P^34^]‐NPY nor the 4‐OHT conjugate **1** without a cleavable linker induced any measurable luciferase activity. These results confirm Y_1_R‐mediated uptake of the conjugates into cells, followed by intracellular cleavage of the linker and subsequent drug release.

Interestingly, **5** exhibited a maximum activity similar to 4‐OHT with an EC_50_ value of 26.3 nM. However, in a control experiment with HEK293 cells that expressed ER*α* and an ER*α* reporter luciferase but not hY_1_R (Figure 4C, Table 2), **5** still induced luciferase expression with an EC_50_ value of 34.8 nM. Since the conjugate cannot be taken up by the [F^7^,P^34^]‐NPY/hY_1_R shuttling pathway in this setup, the linker must already be cleaved in the extracellular space. The released 4‐OHT can then passively diffuse through the membrane. For validation, mass spectrometric studies demonstrate that the self‐immolative diamine linker is cleaved at neutral pH in various cell culture media or Dulbecco's phosphate‐buffered saline (DPBS), independent of prior incubation with cells (see Figure S3 and S4, Supporting Information). Unlike the self‐immolative cleavage with unaltered drug release, the reduced maximum effect of conjugates **2–4** is likely due to nonresidue‐free cleavage from the peptide. Endosomal cathepsin B can cleave the GFLG linker between F and L, as well as the CitVE linker after E.^[^ ^26^ ^]^ The drug is coupled to the peptide by an acetic acid. We assume that the acetic acid moiety remains bound to the drug even after a full linker cleavage. This results in a 4carboxymethoxy‐tamoxifen rather than 4‐OHT. The hydroxyl group of 4‐OHT is crucial for binding to the ligand‐binding domain (LBD) in ER*α*.^[^ ^47^ ^]^ However, a study showed that smaller residues on the hydroxyl group only slightly reduce receptor activation properties.^[^ ^35^ ^]^ One alternative to achieve a stronger intracellular effect is using a 4‐OHT analog. This would allow a temporary coupling to the peptide by a functional group at a different position on the molecule that is less important for ER*α* binding, thereby keeping the critical hydroxyl group for binding unmodified. Despite the discussed limitations, proof of principle for the internalization and intracellular release of 4‐OHT was demonstrated. Conjugate **2** not only showed the highest potency, but also induced the strongest arr3 recruitment and luciferase activity. Therefore, it was selected as a model for further investigations.

### 2.4 Peptide Conjugates Show Excellent Stability in Human Blood Plasma

**Figure 5:**
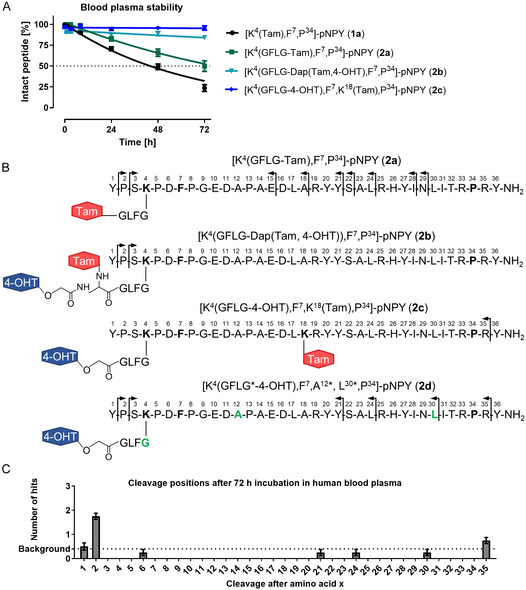
Stability analysis of different Tam‐labeled pNPY analogs and an isotope‐labeled 4‐OHT‐conjugate was performed in human blood plasma. Peptide solutions (10 μM) were incubated at 37 °C and 500 rpm. A) Degradation dynamics of different [F^7^,P^34^]‐pNPY‐based Tamlabelled conjugates. B) Comparison of detected cleavage positions in 4‐OHT‐conjugates using Tam‐ or isotope amino acids in the [F^7^,P^34^]pNPY sequence for peptide‐labeling. C) The measurement of degradation products was conducted using nano‐LC‐electrospray ionization mass spectrometry (ESI‐MS), and subsequent analysis was performed with the software *Proteome Discoverer 2.0*. The occurrence of cleavage sites was detected after a 72 h incubation time. Values represent mean ± SEM of *n *≥ 3 independent experiments. Tam = 6‐carboxytetramethylrhodamine; 4‐OHT = 4hydroxytamoxifen.

For the therapeutic application of 4‐OHT conjugates, it is crucial to validate the stability of the peptides in the human body. In order to investigate the cleavage pattern and degradation dynamics, labels must be attached to the peptides. Common fluorophore labels can alter the degradation dynamics by masking the cleavage positions. Thus, we synthesized different 6‐carboxytetramethylrhodamine (Tam) labeled peptides (**1a**, **2a–2c**, **4a**) and one isotope labeled peptide **2d**. Peptides **2a–d** are all derived from the original peptide **2** and obtained with high purity. To determine the peptide stability, the conjugates were incubated in human blood plasma and samples were collected at designated time points. The prepared samples were analyzed by RP‐HPLC to quantify the degradation (see **Figure** **5**A). The corresponding chromatograms are shown in Figure S5A, Supporting Information.

The [F^7^,P^34^]‐pNPY conjugate **1a** with Tam at Lys^4^ demonstrated the lowest stability with a half‐life of 43.7 h. As previously published, the attachment of linkers and/or drugs to [F^7^,P^34^]‐pNPY, resulting in peptide branching, enhances blood plasma stability.^[^ ^14^ ^]^ The incorporation of the GFLG‐linker into **2a** caused an increase in half‐life to 75.1 h. The type of enzymatic linker does not appear to be a determining factor in this context, as a similar half‐life was determined for the Tam conjugate **4a** with a CitVE‐linker (Figure S5B, Supporting Information). In addition, we compared the cleavage products detected by matrix‐assisted laser desorption/ionization time of flight mass spectrometry (MALDI‐ToF MS) from the eluting peaks in the RP‐HPLC of the 72 h samples (see Figure 5B). In **2a**, several cleavage sites were identified that are primarily located in the N‐terminal or central region of the peptide sequence. Some of these cleavage sites are identical to the previously published cleavage sites of **1a**.^[^ ^48^ ^]^ In particular, the N‐terminal truncation after Tyr^1^ or Pro^2^ by DPP4 was the major product in **2a**.^[^ ^49^ ^,^ ^50^ ^]^ The simultaneous modification of peptides with Tam and 4‐OHT in two different variations led to a considerable enhancement in stability. **2b** exhibited 84.3% intact peptide after 72 h of incubation in human blood plasma, while **2c** showed 95.7% intact peptide under the same conditions. A minimal amount of N‐terminally truncated peptide was observed for conjugate **2b**, and a marginal amount of C‐terminal truncation was measured for conjugate **2c**. Thus, we hypothesize that the attachment of two large, hydrophobic molecules, such as Tam and 4‐OHT, hinders the accessibility of various cleavage sites for peptidases, thereby stabilizing the peptide backbone. Although previous research has demonstrated that fluorophore attachment can have a strong influence on the biochemical properties of small biomolecules such as peptides,^[^ ^48^ ^,^ ^51^ ^,^ ^52^ ^]^ the current study did not observe substantial variations between conjugates **2b** and **2c**. We suggest that the branching of the peptide, initiated by the linker and the drug attachment, results in an enhanced binding to the human serum albumin and ensures a notable increase in stability, thereby negating the impact of the Tam label. To confirm this assumption, we synthesized the isotope‐labeled conjugate **2d**. In general, the incorporation of isotope labels in the peptide does not alter the secondary structure, which allows for more valid information about the peptide stability. Using a mixture of the unlabeled conjugate **2** with conjugate **2d** in a ratio of 2:1 (*n/n*), we were able to distinguish between peptide signals and background, because only hits that were found for both peptides were considered. Using this approach, fewer cleavage sites were identified in conjugate **2d** (see Figure 5C) compared to conjugate **2a**. Despite the challenges of quantifying the measured cleavage products,^[^ ^53^ ^]^ the number of hits strongly indicates the stability or instability of the peptide. In this case, we can assume that conjugate **2d** is highly stable because only a few distinguishable cleavage sites were identified. These cleavage sites are localized in the N‐terminal and C‐terminal region, which correlates with the cleavage sites identified in conjugates **2b** and **2c**. Consequently, we demonstrated the excellent blood plasma stability of the 4‐OHT conjugates with enzymatically cleavable linkers.

### 2.5 Multipayload Conjugation of Peptide with 4‐OHT is Limited

**Figure 6:**
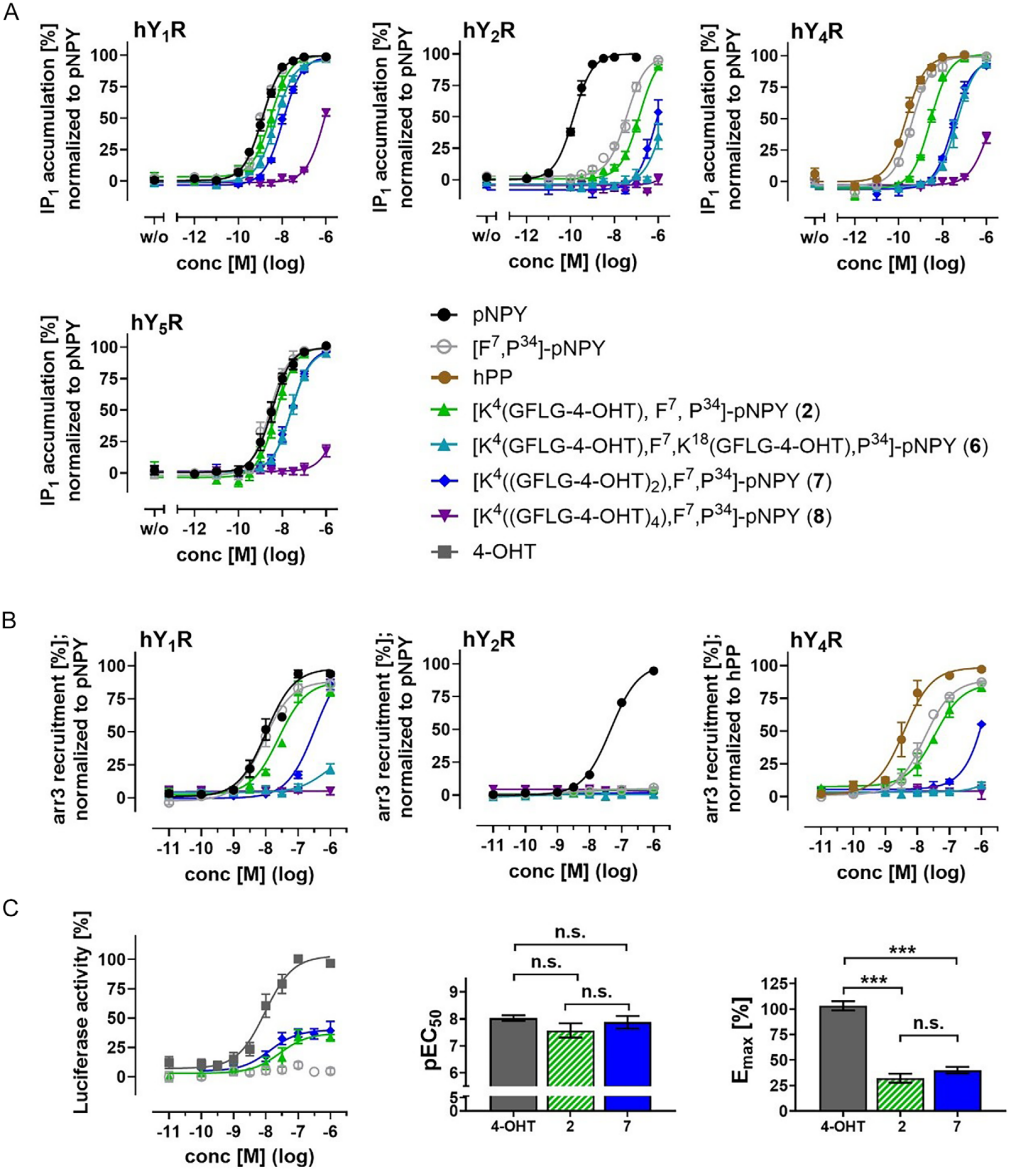
Cellular effects after stimulation with multiply loaded tamoxifen‐conjugates compared to the native ligands, to unmodified [F^7^,P^34^]‐NPY and to a single loaded tamoxifen‐conjugate. A) IP1 accumulation was validated in stably transfected COS‐7‐hY_1/2/4/5_R‐*Δ*6G*α*_qi4‐myr_‐cells. Data are given as mean ± SEM from *n *≥ 2 independent experiments and were performed with technical triplicates. B) Arrestin‐3 (arr3) recruitment to hY_1/2/4_R was measured in a BRET‐based recruitment assay 15 min after stimulation. HEK293 cells were transiently transfected with hY_1/2/4_R‐eYFP and Nluc‐arr3. Data are shown as mean ± SEM from *n *≥ 2 independent experiments, which were done in technical triplicates. C) Intracellular activity of tamoxifen after internalization was investigated with a Luciferase reporter gene assay. Transiently transfected HEK293‐cells expressing hY_1_R‐eYFP, ER*α*pEGFP, and TATA‐luciferase were stimulated with increasing concentration of 4‐OH‐Tmx, [F^7^,P^34^]‐NPY, or peptide‐conjugates for 24 h. Data represent mean ± SEM from *n *≥ 3 independent experiments with at least technical triplicates. Calculated pEC_50_‐ and *E*_max_‐values for the luciferase reporter gene assay are shown as bar graphs. Dunnnet's 1‐ way ANOVA test was used to determine statistical significance; *, *p* < 0.033; **, *p* < 0.002; ***, *p* < 0.001, n.s. = not significant.

We tried to enhance the drug‐related intracellular effect by increasing the payload per shuttled peptide. Compared to [F^7^,P^34^]‐pNPY, which exhibited an EC_50_ value of 1.2 nM, the EC_50_ values measured for conjugates **6** and **7** were 5.0 and 10.8 nM, respectively (see **Figure** **6**A, **Table** **3**). Quadruple peptide loading with 4‐OHT, on the other hand, resulted in a substantial loss of potency, with an almost 1000‐fold higher EC_50_ value (conjugate **8**). At hY_2_R and hY_4_R, the EC_50_ values of the multiple‐loaded conjugates exhibit a greater EC_50_ shift compared to the single‐modified conjugate **2**, suggesting a slightly higher selectivity for hY_1_R. In the arr3 recruitment BRET assay, both **6** and **8** were found to be unable to induce arr3‐mediated receptor internalization (see Figure 6B). A double modification at Lys^4^, as present in **7**, resulted in ≈40‐fold reduction in arr3 recruitment compared to [F^7^,P^34^]‐pNPY. As expected, no conjugate‐induced arr3 recruitment was observed at hY_2_R. Furthermore, at hY_4_R, the potential to recruit arr3 was drastically reduced for **7** and was undetectable for **6** and **8**. The attachment of multiple hydrophobic 4‐OHT molecules likely affects solubility and, consequently, inhibits the receptor binding properties of the peptide. Increased hydrophilicity of the peptides may serve to reduce this problem and allow for the loading of more drug molecules per peptide.^[^ ^54^ ^]^ Nevertheless, to validate a possibly stronger effect, the intracellular release of 4‐OHT after the internalization of **7** was compared with that of **2** (see Figure 6C). However, the analysis did not reveal a significantly better EC_50_ value or a significant increase in the maximum activity.

**Table 3:** Potency of multiply loaded 4‐OHT‐conjugates compared to the native ligands and to unmodified [F^7^,P^34^]‐NPY at different Y receptors. An IP1 accumulation assay for validation of G‐protein activation was conducted in stably transfected COS‐7‐hY_1/2/4/5_R‐Δ6G*α*_qi4‐myr_‐cells. Data were normalized to the corresponding native ligand (NPY for hY_1_R, hY_2_R, hY_5_R, and hPP for hY_4_R). Data are shown as mean ± SEM from *n *≥ 2 independent experiments performed with technical triplicates. For each conjugate, EC_50_‐values and pEC_50 _± SEM are listed in the table.

	EC_50_ values [nM] (pEC_50 _± SEM)						
	IP1 assay				arr3 recruitment BRET		
	hY_1_R	hY_2_R	hY_4_R	hY_5_R	hY_1_R	hY_2_R	hY_4_R
pNPY/ hPP	1.3 (8.9 ± 0.03)	0.1 (9.9 ± 0.04)	0.2 (9.7 ± 0.05)	3.4 (8.5 ± 0.06)	10.1 (8.0 ± 0.08)	45.8 (7.3 ± 0.02)	3.9 (8.4 ± 0.10)
[F^7^,P^34^]‐pNPY	1.2 (8.9 ± 0.04)	34.1 (7.5 ± 0.04)	0.4 (9.4 ± 0.05)	2.6 (8.6 ± 0.07)	8.7 (8.1 ± 0.08)	n. d.	16.9 (7.8 ± 0.08)
2	3.1 (8.5 ± 0.06)	126.6 (6.9 ± 0.04)	2.8 (8.6 ± 0.05)	5.3 (8.3 ± 0.07)	25.0 (7.6 ± 0.12)	n. d.	34.9 (7.5 ± 0.11)
6	5.0 (8.3 ± 0.06)	>1000	45.4 (7.3 ± 0.06)	25.5 (7.6 ± 0.05)	n. d.	n. d.	n. d.
7	10.8 (8.0 ± 0.04)	>800	30.6 (7.51 ± 0.06)	26.0 (7.6 ± 0.05)	336.7 (6.5 ± 0.13)	n. d.	>1000
8	>1000	n. d.	n. d.	n. d.	n. d.	n. d.	n. d.

## 3 Conclusion

4‐OHT has emerged as a promising drug for treating obesity. Transient coupling to a [F^7^,P^34^]‐pNPY peptide shuttle enhances hY_1_R selectivity. In this study, we used a set of cell‐based in vitro assays to validate the hY_1_R‐specific uptake and subsequent intracellular release of the pharmaceutical compound into the cytosol. A luciferase reporter gene assay clearly demonstrated the intracellular activity of 4‐OHT. This is evidenced by the activation of ER*α* by 4‐OHT and subsequent ER*α*‐induced luciferase expression, which could be measured despite the incomplete residue‐free drug release. The conjugate with the GFLG linker was determined to be the optimal proof‐of‐principle model, exhibiting the most promising potency and internalization rates. This peptide‐drug conjugate demonstrated remarkable stability within human blood plasma. Consequently, it provides optimal conditions for further optimization, such as the utilization of 4‐OHT analogs to facilitate drug coupling to the peptide carrier with enhanced synthesis yield and improved intracellular drug activity. Thus, in the next step with improved drug release, the intracellular effect on adipose tissue should be validated with in vivo mice studies.

## 4 Experimental Section

4.1

4.1.1

##### Solid Phase Peptide Synthesis of Peptide Drug Conjugates

PDCs were synthesized by automated and manual SPPS using the fluorenyl methoxycarbonyl/tertbutyl (Fmoc/tBu) strategy in a 15 µmol scale on a NovaSyn TGR R resin (Merck). For automated synthesis in a SYRO II peptide synthesizer (MultiSynTech), all Fmoc‐protected L‐amino acids (Iris Biotech) were dissolved in HOBt (Novabiochem) and diluted in DMF (Biosolve) to a final concentration of 0.3 M each. After swelling of the resin beads in DMF, Fmoc cleavage from the resin was performed with 40% (*v/v*) piperidine (Sigma–Aldrich) in DMF for 3 min and 20% (*v/v*) piperidine in DMF for 10 min afterward. Automated elongation of the peptide was achieved by using 8 equiv. amino acid, DIC (IRIS Biotech), and ethyl cyano(hydroxyimino)acetate (OxymaPure, CEM) for 30 min. Each amino acid coupling was performed twice and under shaking at room temperature. The cycle of Fmoc deprotection and double amino acid coupling was repeated until the desired peptide length was achieved. Fmoc‐L‐2,3‐diaminopropionic acid(Mtt)‐OH (Fmoc‐L‐Dap(Mtt)‐OH, Iris Biotech) and bromoacetic acid (BAA, Sigma–Aldrich) were manually coupled with 5 equiv. amino acid and equimolar amounts of HOBt and DIC for 3‐16 h. Isotope‐labeled amino acids (eurisotop) were manually double‐coupled by using 2 equiv. isotope‐labeled amino acid, with 2 equiv. HOBt and 2 equiv. DIC in DMF overnight. Manual Fmoc deprotection was achieved by double incubation with 20% (*v/v*) piperidine in DMF for 10 min. Methoxytrityl (Mmt) cleavage was conducted by incubation with 2% trifluoroacetic acid (TFA, *v/v*, Merck), 5% triisopropylsilane (TIS, *v/v*, Sigma–Aldrich) in dichloromethane (DCM, Biosolve) for 1 min. After washing with DCM, the reaction was repeated 15 times. 6carboxytetramethylrhodamine (Tam, ChemPep Inc.) was attached to the peptide sequence in a double coupling step using 2 equiv. Tam, 1.9 equiv. *O*‐(7‐azabenzotriazol‐1‐yl)‐*N, N,N′, N′‐*tetramethyluronium hexafluorophosphate (HATU, Sigma–Aldrich), and 2 equiv. *N, N‐*diisopropylethylamine (DIPEA, Sigma–Aldrich) in DMF overnight. 1.5 equiv 4‐OH tamoxifen and 3 equiv. K_2_CO_3_ (Sigma Aldrich) were coupled to the peptide with water‐free THF (Sigma Aldrich) in an argon atmosphere for 3 h at 40 °C under shaking two times. The diamine self‐immolative linker was synthesized in four coupling steps. First, 5 equiv. Fmoc‐Gly‐OH was coupled with equimolar amounts of HOBt and DIC for 6 h. Fmoc was cleaved as described above. 5 equiv. of 6bromohexanoic acid (Merck) and HOBt in DMF were transferred to the resin. Coupling was initiated by adding 5 equiv. DIC and the resin were shaken overnight. In the next step, 5 equiv. N‐Boc‐N‐methylethylenediamine (Fisher Scientific) was coupled with 20 equiv. DIPEA in anhydrous DCM for 4 h. 3 equiv. 4‐OHT and 18 equiv. 4Dimethylaminopyridine (DMAP) was dissolved in anhydrous DCM in a Schlenk flask under nitrogen atmosphere. Afterward, 3 equiv. triphosgene (*v/v*, Sigma–Aldrich) in anhydrous DCM was added dropwise to the prepared 4‐OHT/DMAP mixture. After stirring for 1 h, the resin was added to the solution, followed by another 18 equiv. of DMAP. After 18 h reaction time, the resin was washed with 5x DMF and 5x DCM. After complete synthesis, PDCs were cleaved from the resin using 90% trifluoroacetic acid (TFA, *v/v*, Merck), 7% thioanisole (TA, *v/v*, Sigma–Aldrich), and 3% 2,2′‐(ethylendioxy)‐diethanthiol (DODT, *v/v*, Sigma–Aldrich) for 3 h. Crude peptide was precipitated from ice‐cold diethyl ether (Merck) at −20 °C for at least 1 h, washed with diethyl ether three times and lyophilized. For purification, the peptides were dissolved in 25–35% (*v/v*) acetonitrile (ACN, VWR) in H_2_O. Separation from side‐products was obtained by preparative RP‐HPLC (Hitachi) using the Aeris Peptide XB‐C18 column (250 × 21.2 mm, 100 Å, 5 µm, flow rate 15 mL min^−1^, Phenomenex). A linear gradient of eluent B (0.08% TFA in ACN (v/v)) in eluent A (0.1% TFA in H_2_O (v/v)) increasing by 1% per min over 20–25 min was used. Pure peptide fractions were combined and final purity was confirmed with RP‐HPLC on two different columns (Aeris Peptide 3,6u XB‐C18‐column (250 × 4,6 mm, 100 Å, 3,6 μm, flow rate 1.55 mL min^−1^; Jupiter 4u Proteo 90 Å C‐12‐column (250 × 4,0 mm, 90 Å, 4 μm, flow rate 1 mL min^−1^ or Kinetex Biphenyl (250 × 4.6 mm; 5 µm; 100 Å, flow rate = 1.55 mL min^−1^, Phenomenex). A linear gradient of eluent B in eluent A increasing by 1.25% min^−1^ over 40 min at 40 °C was used and detection occurred at *λ *= 220 nm and *λ *= 280 nm. Peptide identity was validated by MALDI‐ToF MS on an Ultraflex III (Bruker Daltonics) or ESI–MS (Orbitrap Elite, Thermo Fisher Scientific).

##### Investigation of Peptide Stability in Human Blood Plasma

Peptide stability was studied as published recently.^[^ ^48^ ^]^ Human blood plasma was provided by “Institut für Transfusionsmedizin”, Medical Center, Leipzig University and was evaluated by the ethics commission of the Medical Faculty of Leipzig University (“Entwicklung stabilisierter Peptidanaloga für die Wirkstoffforschung”, Aktenzeichen 527/21‐ek). Briefly, peptide solutions in human blood plasma/DPBS (Biowest; 1:1, v/v) were incubated at 37 °C and samples were precipitated in 2× ACN/EtOH (1:1, v/v) at −20 °C overnight. After processing, samples were analyzed by RP‐HPLC using a linear gradient of eluent B (0.1% TFA in H_2_O) in eluent A (0.08% TFA in ACN) over 40 min at 40 °C on a VariTide RPC column (250 × 4.6 mm, 200 Å, 6 μm, flow rate 1 mL min^−1^, Agilent Technologies). Relative amount of intact peptide was calculated by Tam fluorescence intensity (extinction 525 nm, emission 572 nm). With GraphPad Prism 10 the half‐life time was calculated by one the phase decay. Eluting peptides were analyzed by MALDI‐ToF‐MS. Isotope‐labeled peptides were dissolved in H_2_O/20% ACN/0.1% formic acid (FA, *v/v/v*, Sigma ‐Aldrich) after lyophilization, and centrifuged for 5 min at 13,000 × *g*. The supernatant was used for nano‐LC/MS/MS analysis with a nLC1000 UHPLC system (Thermo Fisher Scientific) coupled to the EASY‐Spray ion source of an Orbitrap Elite mass spectrometer (Thermo Fisher Scientific). The measurement and the following data analysis with *Proteome Discoverer 2.0* Software (Thermo Fisher Scientific) were performed as published before.^[^ ^48^ ^]^

##### Cell Culture

All cell lines were cultivated under standard conditions (37 °C, 5% CO_2_ and humified atmosphere) in T75 flasks. After removing old cell media, cells were washed with DPBS (Lonza), detached with trypsin/EDTA (Lonza) and resuspended in fresh cell culture medium. Stably transfected COS‐7 cells expressing hY_1/2/4/5_R and the chimeric G protein G*α*_*Δ*6qi4myr_ were grown in Dulbecco's modified Eagle's medium (DMEM, Lonza) supplemented with 10% heat‐inactivated FBS (Biochrom), 133 µg mL^−1^ hygromycin B (Invivogen) and 1.5 mg mL^−1^ G418 sulfate (Gibco). Nontransfected HEK293 cells were cultivated in DMEM)/Ham's F12 (1:1, v/v, Lonza) with 15% heat‐inactivated FBS.

##### Ca^2+^‐Flux Assay

For quantification of released Ca^2+^‐ions after stimulation, stably transfected COS‐7 cells expressing hY_1/2/4/5_ R and the chimeric G protein G*α*_*Δ*6qi4myr_ were tested in a Ca^2+^‐flux assay. On the first day, cells were seeded in a black, clear‐bottom 96‐well plate (Greiner) and incubated overnight under standard conditions. The next day, Ca^2+^‐flux was analyzed as described previously.^[^ ^55^ ^]^ After incubation of the cells with 50 µl well^−1^ Fluo‐2‐AM solution (2.3 µM Fluo2AM, 0.06% (v/v) Pluronic‐F127 in assay buffer, Abcam) for 1 h, the staining solution was aspirated and replaced by 100 µl well^−1^ assay buffer (20 mM HEPES, 2.5 mM Probenecid in HBSS (Lonza), pH 7.5 at 37 °C). Basal cellular Ca^2+^‐level was measured for 18 s with a Flexstation 3 (Molecular Devices, *λ*_ex_ = 485 nm, *λ*_em_ = 525 nm). Peptide conjugates in different concentrations were added and the Ca^2+^‐response was measured for 36 s. After stimulation of the cells with peptide ligands, the maximal Ca^2+^ response was divided by the basal value and normalized to the top and bottom values of the control peptides NPY for the hY_1/2/5_R and hPP for the hY_4_R. All experiments were performed in technical triplicate and tested at least twice. Nonlinear regression curves were calculated with GraphPad Prism 10.

##### IP1 Accumulation Assay

For receptor activity, intracellular inositol monophosphate (IP1) accumulation was measured with the homogenous time‐resolved fluorescence (HTRF) IP‐One Gq Detection Kit (Cisbio) in stably transfected COS‐7 cells expressing hY_1/2/4/5_R and G*α*_*Δ*6qi4myr_ as reported before.^[^ ^56^ ^]^ Briefly, different cell numbers (hY_1/5_R_G*α*_*Δ*6qi4myr_: 10,000 cells/well, hY_2/4_R_G*α*_*Δ*6qi4myr_: 5000 cells/well) in 20 µL were seeded in white 364‐well plates (Greiner Bio‐One) and maintained overnight under standard conditions. The next day, peptide dilution series were prepared in Hank's balanced salt solution (HBSS, Merck) substituted with 10 mM lithium chloride (Sigma–Aldrich) and 1 mM DMSO. Old cell medium was removed, cells were stimulated with 15 µL well^−1^ of the peptide dilutions and incubated for 45–60 min at standard conditions. Afterward, 3µL well^−1^ of d2‐labeled IP1 acceptor and anti‐IP1 cryptate donor were added and incubated at room temperature for 1 h in the dark. HTRF signals were detected with a Spark plate reader (Tecan Trading AG). Values were calculated by dividing the fluorescence signal of the Förster resonance energy transfer (FRET) acceptor (excitation wavelength (*λ*_ex_) = 320  and 25 nm bandwidth, emission wavelength (*λ*_em_) = 665  and 8 nm bandwidth) by the signal of the FRET donor (*λ*_ex_ = 320 ± 25 nm, *λ*_em_ = 620 ± 10 nm). GraphPad prism 10 was used for data analysis. By generating an IP1 standard curve, concentration response curves within 10% and 90% of the maximal effect were calculated and normalized to the control peptides pNPY for hY_1/2/5_R or hPP for hY_4_R. Each peptide concentration was tested in technical triplicate and was repeated at least three times.

##### Bioluminescence Resonance Energy Transfer (BRET)‐based Arrestin‐3 (arr3) Recruitment Assay

Intracellular arr3 recruitment to the cell membrane was analyzed with a BRET‐based approach, as published before.^[^ ^57^ ^]^ Briefly summarized, HEK293 cells were transiently transfected with 9900 ng hY1R‐eYFP‐pVitro2, hY2ReYFP‐pVitro2, or hY4R‐eYFP‐pVitro2 with 60 ng Nluc‐arr3‐pcDNA in T75 cells culture flasks using MetafectenePro (Biontex Laboratories GmbH) based on the manufacturer's protocol. The next day, cells were seeded in white 96‐well plates (Greiner Bio‐One). Two days after transfection, the medium was replaced with 100 µL well^−1^ BRET buffer (HBSS, 25 mM HEPES, pH 7.3 at 37 °C), and 50 µL well^−1^ coelenterazine‐H (DiscoverX, final concentration of 4.2 µM). Fourfold concentrated peptide dilution series in BRET‐buffer were prepared and 50 µL well^−1^ of the dilutions were added to the cells. Buffer without peptide was used as a control for basal activity. After 15 min of stimulation, the BRET signal was measured with a Tecan Spark plate reader at 37 °C (luminescence filter 400–470 nm and fluorescence filter 505–590 nm) and the ratio of fluorescence and luminescence ratio was calculated as a function of peptide concentration. Buffer control values were subtracted and nonlinear regression curves were calculated using GraphPad Prism 10. The curves were normalized to top and bottom values of the control peptides pNPY for hY_1/2_ R and hPP for hY_4_R. All experiments were performed in technical and biological triplicates.

##### Luciferase Reporter Gene Assay

To validate intracellular release of 4‐OHT after incubation of Y_1_R expressing cells with 4‐OHT‐peptide conjugates, a luciferase reporter gene assay was established. HEK293‐cells were transiently transfected with 0.083 µg/well hY_1_R‐eYFP‐pVitro2, pEGFP‐c1‐ER*α* (kindly provided by Michael Mancini (Addgene plasmid #28230; http://n2t.net/addgene:28230; RRID:Addgene_28230))^[^ ^58^ ^]^ and 3xERE‐Tata‐Luc (kindly provided by Donald McDonnell (Addgene plasmid #11354; http://n2t.net/addgene:11354; RRID:Addgene_11354))^[^ ^59^ ^]^ parallel to cell seeding. For a control experiment without hY_1_R‐expression, pEGFP‐N1 together with pEGFP‐c1‐ER*α* and 3xERETata‐Luc was transfected instead. Lipofectamine2000 (Invitrogen) was used as a transfection reagent according to the manufacturer's protocol. The transfection solution with lipofectamine and the plasmids was carefully mixed with a HEK293 cell suspension (100.000 cells/150 µl). 200 µl/well of the complete transfection suspension was seeded into poly‐D‐lysine‐coated Nunc microwell 96‐well optical bottom plates (Merck). The cells were incubated under standard conditions overnight. Cells were washed with DPBS the next day and phenol red‐free and FBS‐free DMEM was added for starving. After an additional 24 h of incubation at 37 °C, cells were stimulated with differing concentrations between 10^−6^ and 10^−11 ^M of 4‐OH‐Tmx and peptide conjugates and were cultivated in the incubator until measurement the next day. Luciferase activity was detected with ONE Glo Luciferase Assay System (Promega) following the manufacturer's specifications. Each concentration was measured at least in technical triplicates and relative luminescence units (RLU) were obtained by normalization to free 4‐OH‐Tmx. Concentration response curves were calculated with Graph Pad Prism 9.

## Conflict of Interest

The authors declare no conflict of interest.

## Supporting information

Supplementary Material

## Data Availability

The data that support the findings of this study are available from the corresponding author upon reasonable request.

## References

[1] A. Accardo , L. Aloj , M. Aurilio , G. Morelli , D. Tesauro , Int. J. Nanomed. 2014, 9, 1537. 10.2147/IJN.S53593 PMC3970945 24741304

[2] W. Sun , F. Tang , J.‐X. Cui , Z.‐L. Lu , ACS Omega 2020, 5, 31700. 33344822 10.1021/acsomega.0c04213 PMC7745405

[3] B. M. Cooper , J. Iegre , D. H. O.’ Donovan , M.Ölwegård Halvarsson , D. R. Spring , Chem. Soc. Rev. 2021, 50, 1480. 33346298 10.1039/d0cs00556h

[4] K. Sriram , P. A. Insel , Mol. Pharmacol. 2018, 93, 251. 10.1124/mol.117.111062 PMC5820538 29298813

[5] K. Bellmann‐Sickert , A. G. Beck‐Sickinger , Trend Pharmacol. Sci. 2010, 31, 434. 10.1016/j.tips.2010.06.003 20655603

[6] H. Herzog , Eur. J. Pharmacol. 2003, 480, 21. 14623347 10.1016/j.ejphar.2003.08.089

[7] E. Parker , M. van Heek , A. Stamford , Eur. J. Pharmacol. 2002, 440, 173. 12007534 10.1016/s0014-2999(02)01427-9

[8] J. C. Reubi , M. Gugger , B. Waser , J. C. Schaer , Cancer Res. 2001, 61, 4636. 11389101

[9] M. Körner , J. C. Reubi , Peptides 2007, 28, 419. 17223228 10.1016/j.peptides.2006.08.037

[10] S. Amisten , M. Neville , R. Hawkes , S. J. Persaud , F. Karpe , A. Salehi , Pharmacol. Ther. 2015, 146, 61. 25242198 10.1016/j.pharmthera.2014.09.007

[11] M. T. Gericke , J. Kosacka , D. Koch , M. Nowicki , T. Schröder , A. M. Ricken , K. Nieber , K. Spanel‐Borowski , Br. J. Pharmacol. 2009, 157, 620. 19422400 10.1111/j.1476-5381.2009.00164.x PMC2707974

[12] Y. Hua , D. Xie , Y. Zhang , M. Wang , W. Wen , J. Sun , Gene 2023, 888, 147755. 37659596 10.1016/j.gene.2023.147755

[13] K. Tatemoto , M. Carlquist , V. Mutt , Nature 1982, 296, 659. 6896083 10.1038/296659a0

[14] D. Böhme , A. G. Beck‐Sickinger , ChemMedChem 2015, 10, 804. 25914147 10.1002/cmdc.201402514

[15] E.‐M. Jülke , B. Özbay , M. Nowicki , S. Els‐Heindl , K. Immig , K. Mörl , I. Bechmann , A. G. Beck‐Sickinger , ACS Pharmacol. Transl. Sci. 2025, 8, 1168. 40242586 10.1021/acsptsci.5c00082 PMC11997893

[16] S. Wittrisch , N. Klöting , K. Mörl , R. Chakaroun , M. Blüher , A. G. Beck‐Sickinger , Mol. Metab. 2020, 31, 163. 31918918 10.1016/j.molmet.2019.11.009 PMC6931124

[17] M. Schenk , K. Mörl , S. Herzig , A. G. Beck‐Sickinger , J. Pept. Sci. 2024, 30, e3611. 38714526 10.1002/psc.3611

[18] R. M. Söll , M. C. Dinger , I. Lundell , D. Larhammer , A. G. Beck‐Sickinger , Eur. J. Biochem. 2001, 268, 2828. 11358498 10.1046/j.1432-1327.2001.02161.x

[19] V. M. Ahrens , R. Frank , S. Stadlbauer , A. G. Beck‐Sickinger , E. Hey‐Hawkins , J. Med. Chem. 2011, 54, 2368. 21395319 10.1021/jm101514m

[20] V. M. Ahrens , K. B. Kostelnik , R. Rennert , D. Böhme , S. Kalkhof , D. Kosel , L. Weber , M. von Bergen , A. G. Beck‐Sickinger , J. Control Release 2015, 209, 170. 25935706 10.1016/j.jconrel.2015.04.037

[21] D. Zwanziger , I. U. Khan , I. Neundorf , S. Sieger , L. Lehmann , M. Friebe , L. Dinkelborg , A. G. Beck‐Sickinger , Bioconjug. Chem. 2008, 19, 1430. 18572959 10.1021/bc7004297

[22] H. Gicquiaux , S. Lecat , M. Gaire , A. Dieterlen , Y. Mély , K. Takeda , B. Bucher , J.‐L. Galzi , J. Biol. Chem. 2002, 277, 6645. 11741903 10.1074/jbc.M107224200

[23] D. Böhme , A. G. Beck‐Sickinger , J. Pept. Sci. 2015, 21, 186. 25703117 10.1002/psc.2753

[24] V. Omelyanenko , C. Gentry , P. Kopečková , J. Kopeček , Int. J. Cancer 1998, 75, 600. 9466663 10.1002/(sici)1097-0215(19980209)75:4<600::aid-ijc18>3.0.co;2-c

[25] A. Pryyma , S. Gunasekera , J. Lewin , D. M. Perrin , Bioconjug. Chem. 2020, 31, 2685. 33274932 10.1021/acs.bioconjchem.0c00563

[26] Y. Anami , C. M. Yamazaki , W. Xiong , X. Gui , N. Zhang , Z. An , K. Tsuchikama , Nat. Commun. 2018, 9, 2512. 29955061 10.1038/s41467-018-04982-3 PMC6023893

[27] A. R. M. Dias , A. Pina , A. Dean , H.‐G. Lerchen , M. Caruso , F. Gasparri , I. Fraietta , S. Troiani , D. Arosio , L. Belvisi , et al, Chemistry 2019, 25, 1696. 30452790 10.1002/chem.201805447 PMC6471013

[28] J. A. Fuselier , L. Sun , S. N. Woltering , W. A. Murphy , N. Vasilevich , D. H. Coy , Bioorg. Med. Chem. Lett. 2003, 13, 799. 12617894 10.1016/s0960-894x(03)00016-7

[29] G. N. Wade , H. W. Heller , Am. J. Physiol. 1993, 264, R1219. 8322977 10.1152/ajpregu.1993.264.6.R1219

[30] W. J. Wallen , M. P. Belanger , C. Wittnich , J. Nutr. 2001, 131, 2351. 11533278 10.1093/jn/131.9.2351

[31] N. Hesselbarth , C. Pettinelli , M. Gericke , C. Berger , A. Kunath , M. Stumvoll , M. Blüher , N. Klöting , Biochem. Biophys. Res. Commun. 2015, 464, 724. 26164229 10.1016/j.bbrc.2015.07.015

[32] N. Klöting , M. Kern , M. Moruzzi , M. Stumvoll , M. Blüher , Acta Diabetol. 2020, 57, 495. 31909433 10.1007/s00592-019-01468-6 PMC7093358

[33] J. B. Custódio , T. C. Dinis , L. M. Almeida , V. M. Madeira , Biochem. Pharmacol. 1994, 47, 1989. 8010983 10.1016/0006-2952(94)90073-6

[34] S. D. Lyman , V. C. Jordan , Biochem. Pharmacol. 1985, 34, 2787. 4015715 10.1016/0006-2952(85)90580-5

[35] D. D. Yu , J. M. Huss , H. Li , B. M. Forman , Bioorg. Med. Chem. 2017, 25, 1585. 10.1016/j.bmc.2017.01.019 28189393

[36] X. Pedragosa‐Badia , G. R. Sliwoski , E. D. Nguyen , D. Lindner , J. Stichel , K. W. Kaufmann , J. Meiler , A. G. Beck‐Sickinger , J. Biol. Chem. 2014, 289, 5846. 24375409 10.1074/jbc.M113.502021 PMC3937655

[37] A. Wraith , A. Törnsten , P. Chardon , I. Harbitz , B. P. Chowdhary , L. Andersson , L. G. Lundin , D. Larhammar , Genome Res. 2000, 10, 302. 10720571 10.1101/gr.10.3.302 PMC311425

[38] M. M. Berglund , D. A. Schober , M. A. Statnick , P. H. McDonald , D. R. Gehlert , J. Pharmacol. Exp. Ther. 2003, 306, 147. 12665544 10.1124/jpet.103.051227

[39] L. E. Gimenez , S. Babilon , L. Wanka , A. G. Beck‐Sickinger , V. V. Gurevich , Cell. Signal. 2014, 26, 1523. 24686081 10.1016/j.cellsig.2014.03.019 PMC4033671

[40] M. P. Hall , J. Unch , B. F. Binkowski , M. P. Valley , B. L. Butler , M. G. Wood , P. Otto , K. Zimmerman , G. Vidugiris , T. Machleidt , et al, ACS Chem. Biol. 2012, 7, 1848. 22894855 10.1021/cb3002478 PMC3501149

[41] I. Böhme , J. Stichel , C. Walther , K. Mörl , A. G. Beck‐Sickinger , Cell. Signal. 2008, 20, 1740. 18598760 10.1016/j.cellsig.2008.05.017

[42] J. A. Bard , M. W. Walker , T. A. Branchek , R. L. Weinshank , J. Biol. Chem. 1995, 270, 26762. 7592911 10.1074/jbc.270.45.26762

[43] P. Gregor , M. L. Millham , Y. Feng , L. B. DeCarr , M. L. McCaleb , L. J. Cornfield , FEBS Lett. 1996, 381, 58. 8641440 10.1016/0014-5793(96)00067-1

[44] I. Lundell , A. G. Blomqvist , M. M. Berglund , D. A. Schober , D. Johnson , M. A. Statnick , R. A. Gadski , D. R. Gehlert , D. Larhammar , J. Biol. Chem. 1995, 270, 29123. 7493937 10.1074/jbc.270.49.29123

[45] E. Kulkoyluoglu , Z. Madak‐Erdogan , Steroids 2016, 114, 41. 27394959 10.1016/j.steroids.2016.06.007

[46] L. Wang , N. Wang , W. Zhang , X. Cheng , Z. Yan , G. Shao , X. Wang , R. Wang , C. Fu , Signal Transduct. Target Ther. 2022, 7, 48. 10.1038/s41392-022-00904-4 PMC8844085 35165272

[47] A. K. Shiau , D. Barstad , P. M. Loria , L. Cheng , P. J. Kushner , D. A. Agard , G. L. Greene , Cell 1998, 95, 927. 9875847 10.1016/s0092-8674(00)81717-1

[48] A. Kohler , E.‐M. Jülke , J. Stichel , A. G. Beck‐Sickinger , ACS Pharmacol. Transl. Sci. 2024, 7, 3618. 39539263 10.1021/acsptsci.4c00503 PMC11555501

[49] R. Mentlein , Regul. Pept. 1999, 85, 9. 10588446 10.1016/s0167-0115(99)00089-0

[50] J.‐F. Yao , H. Yang , Y.‐Z. Zhao , M. Xue , Curr. Drug Metab. 2018, 19, 892. 29956618 10.2174/1389200219666180628171531

[51] S. F. Hedegaard , M. S. Derbas , T. K. Lind , M. R. Kasimova , M. V. Christensen , M. H. Michaelsen , R. A. Campbell , L. Jorgensen , H. Franzyk , M. Cárdenas , H. M. ørck Nielsen , Sci. Rep. 2018, 8, 6327. 29679078 10.1038/s41598-018-24154-z PMC5910404

[52] H. H. Szeto , P. W. Schiller , K. Zhao , G. Luo , FASEB J. 2005, 19, 118. 15489247 10.1096/fj.04-1982fje

[53] Z. Wang , P.‐K. Liu , L. Li , ACS Meas. Sci. Au. 2024, 4, 315. 39184361 10.1021/acsmeasuresciau.4c00007 PMC11342459

[54] D. J. Worm , P. Hoppenz , S. Els‐Heindl , M. Kellert , R. Kuhnert , S. Saretz , J. Köbberling , B. Riedl , E. Hey‐Hawkins , A. G. Beck‐Sickinger , J. Med. Chem. 2020, 63, 2358. 31589041 10.1021/acs.jmedchem.9b01136

[55] P. Hoppenz , S. Els‐Heindl , M. Kellert , R. Kuhnert , S. Saretz , H.‐G. Lerchen , J. Köbberling , B. Riedl , E. Hey‐Hawkins , A. G. Beck‐Sickinger , J. Org. Chem. 2020, 85, 1446. 31813224 10.1021/acs.joc.9b02406

[56] T. Tang , Q. Tan , S. Han , A. Diemar , K. Löbner , H. Wang , C. Schüß , V. Behr , K. Mörl , M. Wang , X. Chu , C. Yi , M. Keller , J. Kofoed , S. Reedtz‐Runge , A. Kaiser , A. G. Beck‐Sickinger , Q. Zhao , B. Wu , Sci. Adv. 2022, 8, eabm1232. 35507650 10.1126/sciadv.abm1232 PMC9067930

[57] T. F. Fischer , A. S. Czerniak , T. Weiß , T. Zellmann , L. Zielke , S. Els‐Heindl , A. G. Beck‐Sickinger , Cancers 2021, 13, 3788. 34359687 10.3390/cancers13153788 PMC8345219

[58] D. L. Stenoien , M. G. Mancini , K. Patel , E. A. Allegretto , C. L. Smith , M. A. Mancini , Mol. Endocrinol. 2000, 14, 518. 10770489 10.1210/mend.14.4.0436

[59] J. M. Hall , D. P. McDonnell , Endocrinology 1999, 140, 5566. 10579320 10.1210/endo.140.12.7179

