# Supplementary material for: Dual Targeting Approach Using 4‐Hydroxytamoxifen Neuropeptide Y Conjugates for Selective Addressing of Adipose Tissue
Source: ChemMedChem. 2025 Oct 28;20(24):e202500668. doi: 10.1002/cmdc.202500668 (PMC12711160; doi:10.1002/cmdc.202500668)
Supplement: Supplementary file 1 — Supplementary Material [file CMDC-20-e202500668-s001.pdf]

Experimental Supporting Information

Dual targeting approach using 4-hydroxytamoxifen neuropeptide Y conjugates for selective addressing of adipose tissue

Anna Kohler, Eva-Maria Jülke, Luke C. Darveniza, Jan Stichel and Annette G. Beck-Sickinger*

A. Kohler, E.-M. Jülke, J. Stichel, A. G. Beck-Sickinger
Faculty of Life Sciences, Institute of Biochemistry
Leipzig University
04103 Leipzig, Germany
E-mail: abeck-sickinger@uni-leipzig.de

L. C. Darveniza
Molecular Imaging and Therapy Research Unit (MITRU)
South Australian Health and Medical Research Institute (SAHMRI)
2 North Terrace, Adelaide SA 5000, Australia

Table S1: Analytical characterization of synthesized peptides. Peptide identity was verified by comparison of calculated monoisotopic mass (M_{mono}) and detected mass (M_{obs}) by (a) electrospray ionization-orbitrap mass spectrometry, (b) matrix assisted laser desorption ionization-time of flight mass spectrometry or (c) electrospray ionization-high capacity ion trap mass spectrometry. Compound purity and retention time (t_R) was analyzed by reversed phase-high performance liquid chromatography using an linear gradients of 20-70% (*v/v*) eluent B in eluent A over 40 min on (d) Aeris Peptide XB-C18 (250 × 4.6 mm, 3.6 µm, 100 Å, flow rate = 1.55 ml/min), (e) Jupiter Proteo (250 × 4.6 mm; 4 µm; 90 Å, flow rate = 0.6 ml/min) or (f) Kinetex Biphenyl (250 × 4.6 mm; 5 µm; 100 Å, flow rate = 1.55 ml/min). Abbreviations: 4-OHT = 4-hydroxytamoxifen**;** Cit = citrulline; Dap = diaminopropionic acid**;** Tam = 6-carboxytetramethylrhodamine.

	**Peptide**	**Sequence**	**Mass spectrometry**		**t_R [min]**		**Purity [%]**
			**M_{mono} [Da]**	**M_{obs} [Da]**	**Column 1**	**Column 2**	
**Control peptides**	pNPY	YPSKPDNPGEDAPAEDLARYYSALRHYINLITRQRY-NH_2	4251.1	$[M+4H]^{4+}$: 1063.8[a]<br>$[M+5H]^{5+}$: 851.2[a]<br>$[M+6H]^{6+}$: 709.5[a]<br>$[M+7H]^{7+}$: 608.6[a]	15.3[d]	17.1[e]	> 98
	hPP	APLEPVYPGDNATPEQMAQYAADLRRYINMLTRPRY-NH_2	4179.1	$[M+3H]^{3+}$: 1394.7[a]<br>$[M+4H]^{4+}$: 1046.3[a]<br>$[M+5H]^{5+}$: 834.2[a]	15.6[d]	17.4[e]	> 95
	[F^{7},P^{34}]-pNPY	YPSKPDFPGEDAPAEDLARYYSALRHYINLITRPRY-NH_2	4253.1	$[M+1H]^{+}$: 4254.2[b]<br>$[M+2H]^{2+}$: 2127.6[b]	15.1[d]	17.1[e]	> 98
**4-OHT-coupled peptide conjugates**	[K^{4}(4-OHT),F^{7},P^{34}]-pNPY<br>(**1**)	YPSK(**$COCH_2$**-4-OHT)PD<br>FPGEDAPAEDLARYYSALRHYINLITRPRY-NH_2	4680.4	$[M+4H]^{4+}$: 1171.8[a]<br>$[M+5H]^{5+}$: 937.5[a]<br>$[M+6H]^{6+}$: 781.4[a]<br>$[M+7H]^{7+}$: 669.9[a]	1: 20.3[d]<br>2: 20.6[d]	1: 21.0[f]<br>2: 21.2[f]	> 95
	[K^{4}(GFLG-4-OHT),F^{7},P^{34}]-pNPY<br>(**2**)	YPSK(GFLG-**$COCH_2$**-4-OHT)PDFPGED<br>APAEDLARYYSALRHYINLITRPRY-NH_2	5054.6	$[M+5H]^{5+}$: 1012.5[a]<br>$[M+6H]^{6+}$: 843.8[a]<br>$[M+7H]^{7+}$: 723.5[a]	1: 21.6[d]<br>2: 21.8[d]	1: 22.3[f]<br>2: 22.6[f]	> 95
	[K^{4}(CitV-4-OHT),F^{7},P^{34}]-pNPY<br>(**3**)	YPSK(CitV-**$COCH_2$**-4-OHT)PDFPGEDAPAEDLA<br>RYYSALRHYINLITRPRY-NH_2	4936.5	$[M+5H]^{5+}$: 988.3[c]<br>$[M+6H]^{6+}$: 824.0[c]<br>$[M+7H]^{7+}$: 706.5[c]<br>$[M+8H]^{8+}$: 618.3[c]	1: 20.0[d]<br>2: 20.3[d]	1: 21.3[e]<br>2: 21.4[e]	> 95
	[K^{4}(CitVE-4-OHT),F^{7},P^{34}]-pNPY<br>(**4**)	YPSK(CitVE-**$COCH_2$**-4-OHT)PDFPGEDAPAEDLA<br>RYYSALRHYINLITRPRY-NH_2	5065.6	$[M+5H]^{5+}$: 1014.4[c]<br>$[M+6H]^{6+}$: 845.3[c]<br>$[M+7H]^{7+}$: 724.9[c]<br>$[M+8H]^{8+}$: 634.5[c]	1: 19.6[d]<br>2: 19.9[d]	1: 21.1[e]<br>2: 21.2[e]	> 95
	[K^{4}(Diamine-4-OHT),F^{7},P^{34}]-pNPY<br>(**5**)	YPSK($C_{12}H_{22}N_3O_3$-4-OHT)PD<br>FPGEDAPAEDLARYYSALRHYINLITRPRY-NH_2	4893.5	$[M+6H]^{6+}$: 816.9[a]<br>$[M+7H]^{7+}$: 700.4[a]<br>$[M+8H]^{8+}$: 612.7[a]	17.7[d]	19.6[e]	> 88

**Multiple 4-OHT-coupled peptide conjugates**	[K^4(GFLG-4-OHT),F^7,K^{18}(GFLG-4-OHT),P^{34}]-pNPY<br>(**6**)	YPSK(GFLG-**$COCH_2$**-4-OHT)PDFPGEDAPAEDLK<br>(GFLG-**$COCH_2$**-4-OHT)RYYSALRHYINLITRPRY-NH_2	5913.0	$[M+5H]^{5+}$: 1184.2[a]<br>$[M+6H]^{6+}$: 987.0[a]<br>$[M+7H]^{7+}$: 846.0[a]<br>$[M+8H]^{8+}$: 740.5[a]	1: 24.2[d]<br>2: 24.4[d]<br>3: 24.6[d]	25.3[e]	> 92
	[K^4(GFLG-4-OHT)$_2$,F^7,P^{34}]-pNPY<br>(**7**)	YPSK(EG$_3$-DAP(GFLG-**$COCH_2$**-4-OHT)$_2$PD<br>FPGEDAPAEDLARYYSALRHYINLITRPRY-NH_2	6145.1	$[M+5H]^{5+}$: 1231.4[a]<br>$[M+6H]^{6+}$: 1026.2[a]<br>$[M+7H]^{7+}$: 879.6[a]<br>$[M+8H]^{8+}$: 769.2[a]	1: 24.3[d]<br>2: 24.5[d]<br>3: 24.7[d]	26.2[e]	> 95
	[K^4(GFLG-4-OHT)$_4$,F^7,P^{34}]-pNPY<br>(**8**)	YPSK(EG$_3$-DAP(DAP(GFLG-**$COCH_2$**-4-OHT)$_2$)$_2$PD<br>FPGEDAPAEDLARYYSALRHYINLITRPRY-NH_2	7920.1	$[M+7H]^{7+}$: 1133.0[a]<br>$[M+8H]^{8+}$: 991.5[a]<br>$[M+9H]^{9+}$: 881.5[a]<br>$[M+10H]^{10+}$: 793.3[a]	1: 30.4[d]<br>2: 30.6[d]<br>3: 30.8[d]<br>4: 31.0[d]	31.4[e]	> 90
**Peptides for stability tests**	[K^4(Tam),F^7,P^{34}]-pNPY<br>(**1a**)	YPSK(Tam)PDFPGEDAPAEDLARYYSALRHYINLITRPRY-NH_2	4665.3	$[M+4H]^{4+}$: 1167.6[c]<br>$[M+5H]^{5+}$: 934.3[c]<br>$[M+6H]^{6+}$: 778.8[c]<br>$[M+7H]^{7+}$: 667.7[c]<br>$[M+8H]^{8+}$: 584.4[c]	17.1[d]	20.0[f]	> 95
	[K^4(GFLG-Tam),F^7,P^{34}]-pNPY<br>(**2a**)	YPSK(GLFG-Tam)PD<br>FPGEDAPAEDLARYYSALRHYINLITRPRY-NH_2	5039.5	$[M+1H]^{+}$: 5040.6[b]<br>$[M+2H]^{2+}$: 2520.5[b]<br>$[M+3H]^{3+}$: 1680.6[b]	18.7[d]	21.7[f]	> 95
	[K^4(GFLG-Dap(Tam,4-OHT),F^7,P^{34}]-pNPY<br>(**2b**)	YPSK(GFLG-Dap(Tam,**$COCH_2$**-4-OHT)PD<br>FPGEDAPAEDLARYYSALRHYINLITRPRY-NH_2	5552.8	$[M+1H]^{+}$: 5553.9[b]<br>$[M+2H]^{2+}$: 2778.1[b]	1: 21.5[d]<br>2: 21.7[d]	1: 20.8[f]<br>2: 21.0[f]	> 92
	[K^4(GFLG-4-OHT),F^7,K^{18}(Tam),P^{34}]-pNPY<br>(**2c**)	YPSK(**$COCH_2$**-4-OHT)PD<br>FPGEDAPAEDLK(Tam)RYYSALRHYINLITRPRY-NH_2	5523.8	$[M+5H]^{5+}$: 1106.4[a]<br>$[M+6H]^{6+}$: 922.3[a]<br>$[M+7H]^{7+}$: 790.4[a]<br>$[M+8H]^{8+}$: 691.7[a]	1: 20.7[d]<br>2: 21.0[d]	1: 22.5[e]<br>2: 22.9[e]	> 90
	[K^4(GFLG*-4-OHT),F^7,A^{12}*,L^{30}*,P^{34}]-pNPY<br>(**2d**)	YPSK(GFL-G($^{13}C_2$,^{15}N)- **$COCH_2$**-4-OHT)PDFPG<br>EDAPA($^{13}C_3$,^{15}N)EDLARYYSALRHYINL($^{13}C_6$,^{15}N)ITRPRY-NH_2	5068.6	$[M+4H]^{4+}$: 1269.2 [a]<br>$[M+5H]^{5+}$: 1015.1[a]<br>$[M+6H]^{6+}$: 845.9[a]<br>$[M+7H]^{7+}$: 725.2[a]	1: 20.5[d]<br>2: 20.7[d]	1: 22.3[e]<br>2: 22.4[e]	> 92
	[K^4(CitVE-Tam),F^7,P^{34}]-pNPY<br>(**4a**)	YPSK(CitVE-Tam)PD<br>FPGEDAPAEDLARYYSALRHYINLITRPRY-NH_2	5050.5	$[M+1H]^{+}$: 5051.4[b]<br>$[M+2H]^{2+}$: 2526.1[b]	17.0[d]	18.5[e]	> 95

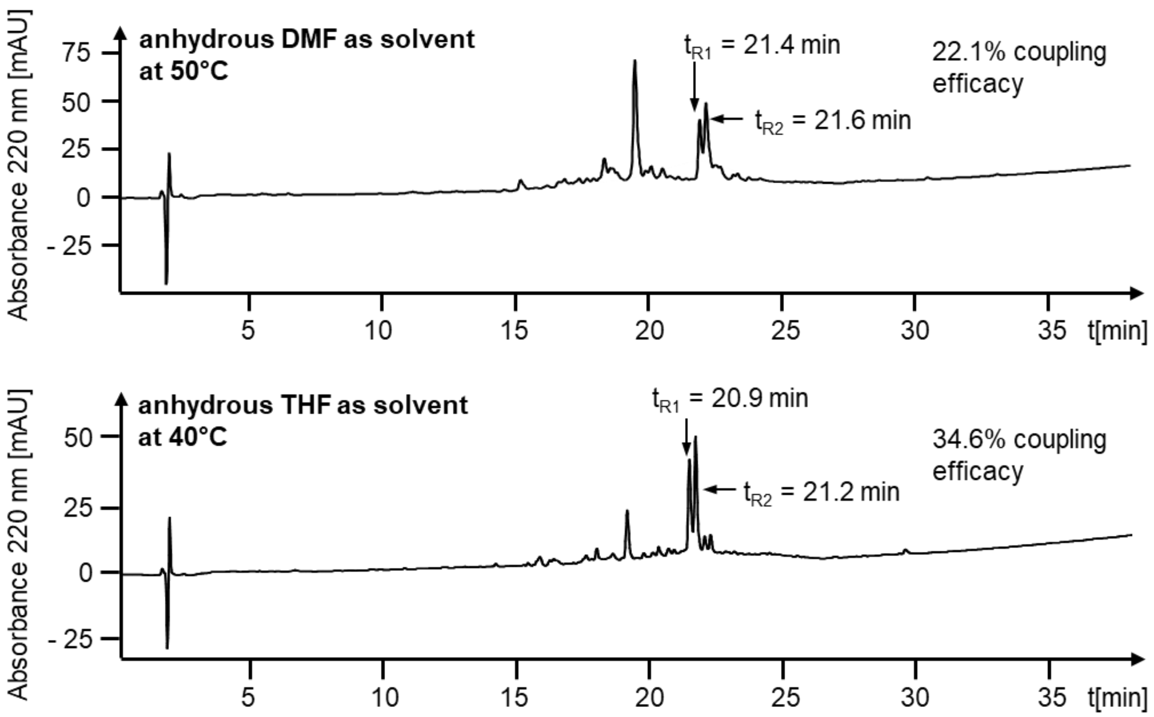

Figure S1: Analytical RP-HPLC was conducted after 4-OHT-coupling using an Aeris Peptide XB-C18 column (250 × 4.6 mm, 3.6 µm, 100 Å, flow rate = 1.55 ml/min) with a linear gradient of 20-70% (v/v) eluent B in eluent A within 40 min. [A] Drug coupling was performed in anhydrous DMF at 50°C using 1.5 eq. 4-OHT and 3 eq. K_2CO_3. [B] Drug coupling was carried out in anhydrous THF at 40°C using 1.5 eq. 4-OHT and 3 eq. K_2CO_3.

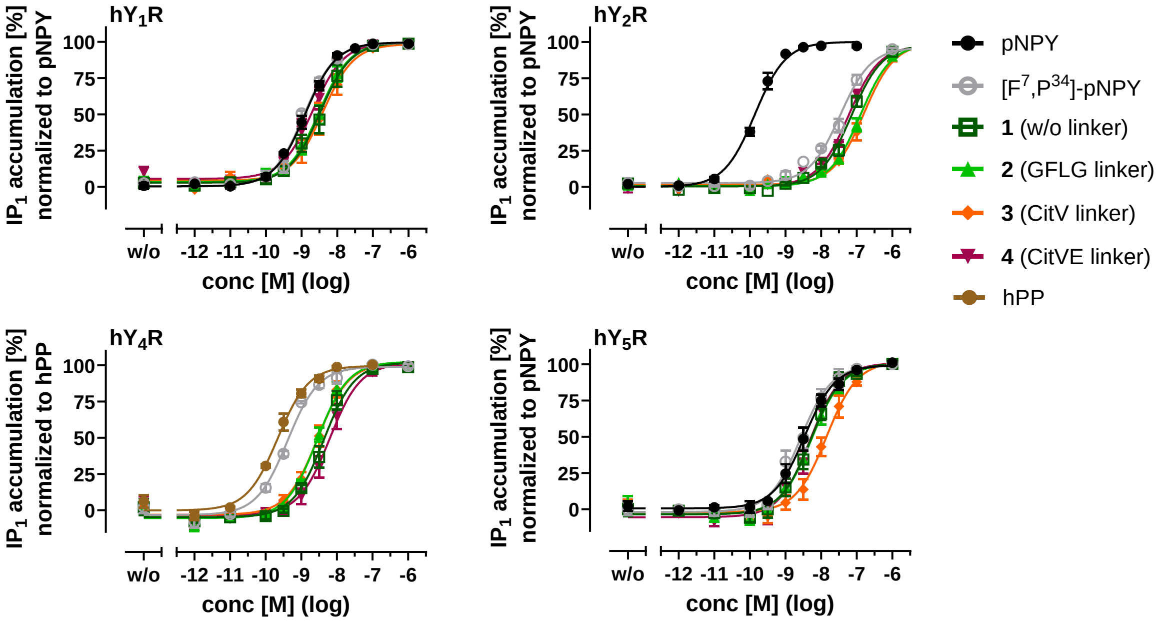

	EC50 values [nM] (pEC50 ± SEM)			
	**hY1R**	**hY2R**	**hY4R**	**hY5R**
**pNPY/ hPP**	1.3 (8.9 ± 0.03)	0.1 (9.9 ± 0.04)	0.2 (9.7 ± 0.05)	3.4 (8.5 ± 0.06)
**[F7,P34]-pNPY**	1.2 (8.9 ± 0.04)	34.1 (7.5 ± 0.04)	0.4 (9.4 ± 0.05)	2.6 (8.6 ± 0.07)
**1** (w/o linker)	3.3 (8.5 ± 0.07)	70.5 (7.2 ± 0.04)	4.3 (8.4 ± 0.06)	5.2 (8.3 ± 0.06)
**2** (GFLG linker)	3.1 (8.5 ± 0.06)	126.6 (6.9 ± 0.04)	2.8 (8.6 ± 0.05)	5.3 (8.3 ± 0.07)
**3** (CitV linker)	3.9 (8.4 ± 0.08)	157.0 (6.8 ± 0.05)	2.9 (8.5 ± 0.06)	14.1 (7.9 ± 0.07)
**4** (CitVE linker)	2.1 (8.7 ± 0.07)	58.5 (7.2 ± 0.03)	6.2 (8.2 ± 0.08)	4.9 (8.3 ± 0.07)

Figure S2: Receptor activation potential of 4-OHT-conjugates compared to the native ligands and unmodified [F^7,P^{34}]-NPY. In stably transfected COS-7-$hY_{1/2/4/5}$R-Δ6$G\alpha_{qi4\text{-}myr}$-cells, G-protein activation was tested using IPone accumulation assay. Sigmoidal curves were achieved by stimulation with increasing peptide concentrations. Data were normalized to the corresponding native ligand (NPY for hY_1R, hY_2R, hY_5R, hPP for hY_4R). Data are shown as mean ± SEM from n ≥ 3 independent experiments performed with technical triplicates. For each conjugate, EC_{50}-values and pEC50 ± SEM are listed in the table.

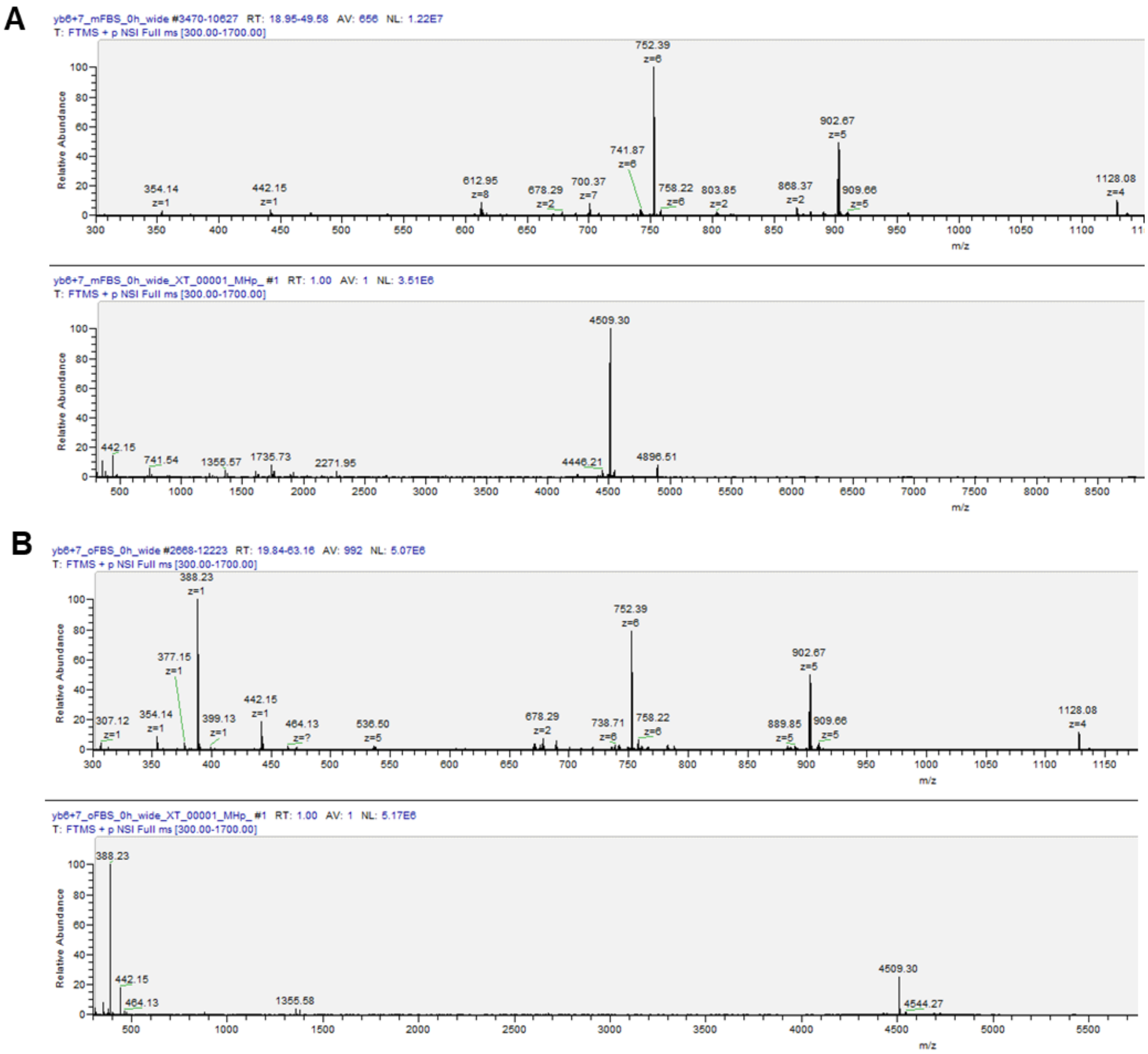

Figure S3: Stability of [K⁴(Diamine-4-OHT),F⁷,P³⁴]-pNPY (**5**) in different solvents was verified with electrospray ionization-orbitrap mass spectrometry. [A] DMEM/Ham's with 15% FBS, pH = 8.01, [B] DMEM/Ham's w/o FBS, pH = 7.04, MW_{mono} of intact peptide = 4893.5 Da, $MW_{average}$ = 4896.5 Da; MW_{mono} of peptide without 4-OHT= 4506.3 Da, $MW_{average}$ = 4509.0 Da.

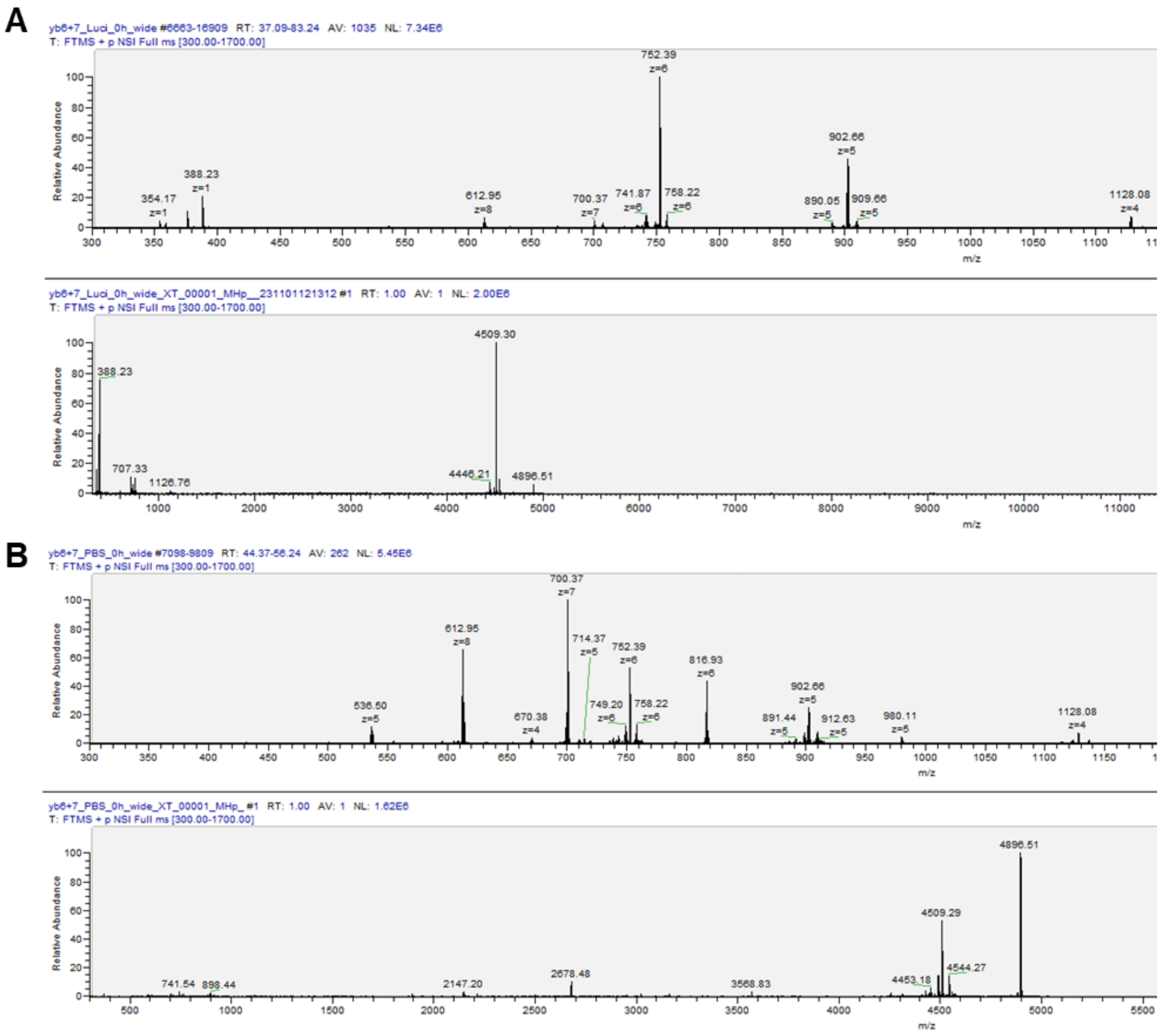

Figure S4: Stability of [K^4(Diamine-4-OHT),F^7,P^{34}]-pNPY (5) in different solvents was verified with electrospray ionization-orbitrap mass spectrometry. [A] luciferase reporter gene assay medium (DMEM/Ham‘s w/o phenolred, w/o FBS, pH = 7.41), [B] DPBS, pH = 7.4. MW_{mono} of intact peptide = 4893.5 Da, $MW_{average}$ = 4896.5 Da; MW_{mono} of peptide without 4-OHT = 4506.3 Da, $MW_{average}$ = 4509.0 Da.

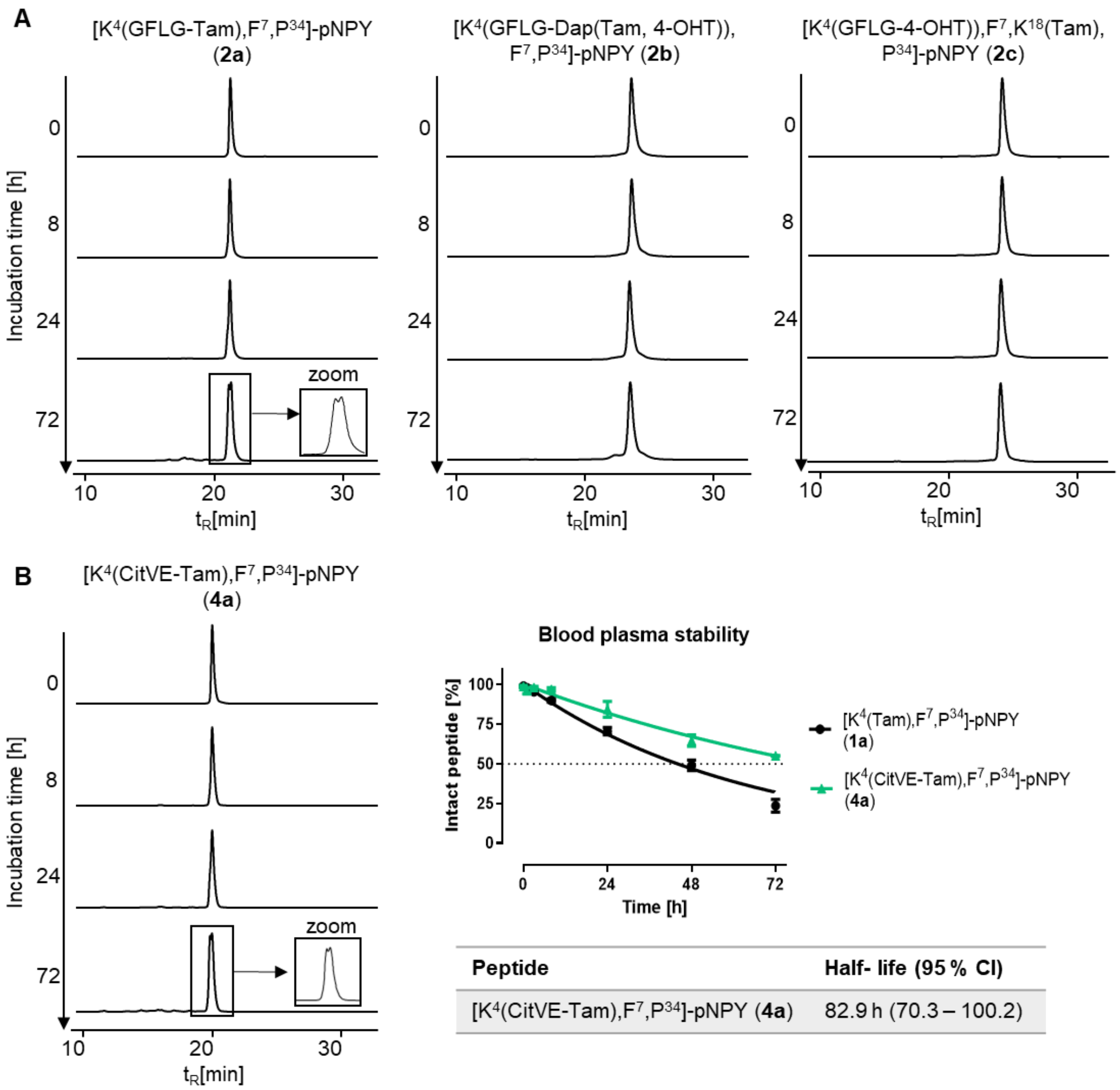

Peptide	Half- life (95 % CI)
[K^4(CitVE-Tam),F^7,P^{34}]-pNPY (**4a**)	82.9 h (70.3 – 100.2)

Figure S5: [A] Degradation of Tam-labelled pNPY analogues with GFLG linker was validated by RP-HPLC with fluorescence detection. [B] RP-HPLC degradation analysis of a Tam-labelled analogue with CitVE linker. The amount of intact peptide was quantified by area under the curve (mean ± SEM of n ≥ 3 independent experiments).
